# KPNB1 modulates the Machado–Joseph disease protein ataxin-3 through activation of the mitochondrial protease CLPP

**DOI:** 10.1007/s00018-022-04372-5

**Published:** 2022-07-06

**Authors:** Mahkameh Abeditashi, Jonasz Jeremiasz Weber, Priscila Pereira Sena, Ana Velic, Maria Kalimeri, Rana Dilara Incebacak Eltemur, Jana Schmidt, Jeannette Hübener-Schmid, Stefan Hauser, Boris Macek, Olaf Riess, Thorsten Schmidt

**Affiliations:** 1 Institute of Medical Genetics and Applied Genomics, University of Tübingen, 72076 Tübingen, Germany; 2 Centre for Rare Diseases, University of Tübingen, 72076 Tübingen, Germany; 3 Graduate Training Centre of Neuroscience, University of Tübingen, 72076 Tübingen, Germany; 4 Department of Human Genetics, Ruhr University Bochum, 44801 Bochum, Germany; 5 Proteome Center Tübingen, University of Tübingen, 72076 Tübingen, Germany; 6 German Center for Neurodegenerative Diseases (DZNE), 72076 Tübingen, Germany; 7 Department of Neurology and Hertie Institute for Clinical Brain Research, University of Tübingen, 72076 Tübingen, Germany

**Keywords:** Ataxin-3, Spinocerebellar ataxia type 3, Polyglutamine diseases, Karyopherins, Mitochondrial protease CLPP, Proteolysis

## Abstract

Machado–Joseph disease (MJD) is characterized by a pathological expansion of the polyglutamine (polyQ) tract within the ataxin-3 protein. Despite its primarily cytoplasmic localization, polyQ-expanded ataxin-3 accumulates in the nucleus and forms intranuclear aggregates in the affected neurons. Due to these histopathological hallmarks, the nucleocytoplasmic transport machinery has garnered attention as an important disease relevant mechanism. Here, we report on MJD cell model-based analysis of the nuclear transport receptor karyopherin subunit beta-1 (KPNB1) and its implications in the molecular pathogenesis of MJD. Although directly interacting with both wild-type and polyQ-expanded ataxin-3, modulating KPNB1 did not alter the intracellular localization of ataxin-3. Instead, overexpression of KPNB1 reduced ataxin-3 protein levels and the aggregate load, thereby improving cell viability. On the other hand, its knockdown and inhibition resulted in the accumulation of soluble and insoluble ataxin-3. Interestingly, the reduction of ataxin-3 was apparently based on protein fragmentation independent of the classical MJD-associated proteolytic pathways. Label-free quantitative proteomics and knockdown experiments identified mitochondrial protease CLPP as a potential mediator of the ataxin-3-degrading effect induced by KPNB1. We confirmed reduction of KPNB1 protein levels in MJD by analyzing two MJD transgenic mouse models and induced pluripotent stem cells (iPSCs) derived from MJD patients. Our results reveal a yet undescribed regulatory function of KPNB1 in controlling the turnover of ataxin-3, thereby highlighting a new potential target of therapeutic value for MJD.

**Supplementary Information:**

The online version contains supplementary material available at 10.1007/s00018-022-04372-5.

## Introduction

Dysfunction in protein homeostasis commonly results in the accumulation of aberrant proteins as toxic aggregates in the cells. Particularly, post-mitotic cells such as neurons are the most affected ones [1]. The formation of intraneuronal proteinaceous inclusions is a histopathological hallmark of polyglutamine (polyQ) diseases comprising Huntington's disease (HD), spinobulbar muscular atrophy (SBMA), dentatorubral–pallidoluysian atrophy (DRPLA), and six types of spinocerebellar ataxias (SCAs). Those inclusions are the consequence of a dynamic expansion of a polyQ tract encoded by a CAG trinucleotide repeat in the respective causative genes [2, 3]. The aggregation propensity of disease proteins depends on the polyQ repeat length, with longer tracts tending to accumulate more intensely [4]. The resulting intracellular aggregates are mainly composed of polyQ-expanded proteins, but recruit molecular chaperones, ubiquitin, proteasome subunits, and transcription factors as well, which may contribute to neuronal dysfunction and death [5–8]. PolyQ diseases are mainly late-onset, relentlessly progressive, and give rise to the neurological and physical impairments in patients [9].

Machado–Joseph disease (MJD), also known as spinocerebellar ataxia type 3 (SCA3), is the most prevalent type of SCA. It is caused by a CAG repeat expansion in the coding region of the *ATXN3/MJD1* gene leading to an elongation of the polyQ tract in the C-terminus of the ataxin-3 protein [10–12]. Normal individuals carry 12–44 CAG repeats, while in MJD patients the expanded repeats range between 61 and 87 CAGs [13]. Ataxin-3 is a deubiquitinating enzyme (DUB) proposed to be implicated in the proteasomal degradation of ubiquitinated proteins [14]. It has two structural features including the N-terminal Josephin domain and the C-terminus which contains two or three ubiquitin interacting motifs (UIMs) flanking the polyQ tract [15]. Despite a mass of significant knowledge, the exact physiological mechanism by which polyQ-expanded ataxin-3 elicits neurotoxicity is still far from clear. The expansion of the polyQ tract evokes the formation of protein aggregates, susceptibility to proteolysis, destabilization of functional conformation, leading to abnormal protein–protein interactions, and ultimately neuronal death [16]. Proteolytic processing of ataxin-3 is thought to generate aggregation-prone fragments, which favors cytotoxicity [17, 18]. Despite predominantly cytoplasmic localization of ataxin-3 [19], several lines of evidence point to the nucleus as the preferential site of aggregation, toxicity, and pathology of this polyQ-expanded protein [20, 21]. Indeed, nuclear localization of ataxin-3 was shown to play a pivotal role in the manifestation of symptoms [22].

Nucleocytoplasmic transport involves the recognition of nuclear localization signals (NLSs) or nuclear export signals (NESs) of cargos by nuclear transport receptors referred to as karyopherins. The classical import pathway is mediated by the karyopherin beta family (KPNBs) that binds to the cargos directly or through adaptor proteins, the karyopherin alpha family (KPNAs) [23, 24]. Ataxin-3 possesses a putative NLS and two NESs which might be involved in its nuclear translocation [25, 26]. Nonetheless, the molecular mechanisms implicated in the nucleocytoplasmic transport of ataxin-3 and their contribution to the pathogenesis of MJD are not fully understood. Previously, our group reported that karyopherin subunit alpha-3 (KPNA3) controls the localization of ataxin-3 and is a key player in the pathogenesis and progression of MJD [27]. Importantly, KPNA3 functions as an adaptor protein for karyopherin subunit beta-1 (KPNB1) in the nuclear transport of various cargos, raising the possibility that KPNB1 could be implicated in the localization and modulation of ataxin-3 as well.

Herein, we report that overexpression and knockdown of KPNB1 did not alter the intracellular localization of ataxin-3, but surprisingly influenced its soluble protein levels and aggregate formation by a proteolytic event independent of known MJD-associated proteolytic pathways. Label-free quantitative proteomics-based pathway analysis of cells overexpressing ataxin-3 and KPNB1 suggested an activation of the ATP-dependent Clp protease proteolytic subunit (CLPP) upon KPNB1 overexpression. Consequently, CLPP knockdown counteracted the KPNB1 overexpression-dependent reduction of ataxin-3 levels, while CLPP overexpression enhanced the KPNB1-mediated ataxin-3 lowering. Additionally, we demonstrated decreased KPNB1 protein levels in two different MJD transgenic mouse models and a reduction of both KPNB1 and CLPP protein levels in induced pluripotent stem cells (iPSCs) of MJD patients. Overall, our findings provide evidence that modulation of soluble and aggregated ataxin-3 by KPNB1 is mediated by an activation of the mitochondrial protease CLPP, highlighting a promising new mechanism for targeting polyQ-expanded disease protein levels.

## Materials and methods

### Mouse housing and tissue sampling

Wild-type mice as well as YAC transgenic mice [28], and CaMKII/MJD77 transgenic mice previously generated in our lab [29], were housed under standard conditions and a 12 h light–dark cycle with 50–55% relative air humidity. Mice were sacrificed using CO_2_ inhalation and brains were immediately separated on ice, snap-frozen in liquid nitrogen, and stored at −80 °C for further analysis.

### Expression constructs and esiRNAs

All plasmids were extracted from DH5α or SURE *E. coli* using the QIAprep Spin Miniprep Kit (QIAGEN, Hilden, Germany) according to the manufacturer’s instructions. Overexpression of ataxin-3 for western blot analysis, nucleocytoplasmic fractionation, and filter retardation assays was carried out using the vector pcDNA3.1/Xpress (Thermo Fisher Scientific, Karlsruhe, Germany) containing full-length human ataxin-3 (UniProt ID: P54252-2) with 15, 77, or 148 glutamines. Empty pcDNA3.1/Xpress vector was used as control. Protein stability analysis was performed using the Tet-off system including pTRE responder containing human ataxin-3 with 15 or 77 glutamines and pTET-RCA2 promoter constructs in a 1:1 ratio [30]. pEGFP-N2 constructs (Clontech, Mountain View, CA, US) containing full-length human ataxin-3 with 15 or 148 glutamines were applied for fluorescence microscopy analysis. pGEX-6P-1 constructs (GE Healthcare, Munich, Germany) carrying human ataxin-3 with 15 or 77 glutamines were used to purify GST-ataxin-3, and an empty pGEX-6P-1 vector served as a control. All ataxin-3 constructs contained isoform 3c. pCMV6-XL5 and pCMV6-Entry/myc-DDK vectors (both OriGene Technologies, Rockville, MD, US) were used for overexpressing human KPNB1 and human CLPP, respectively. Endoribonuclease-prepared siRNA (esiRNA) against human KPNB1 (MISSION esiRNA EHU043151, Sigma-Aldrich, St. Louis, MO, US) and against human CLPP (MISSION esiRNA EHU011941, Sigma-Aldrich) were applied to knockdown endogenous KPNB1 and CLPP, respectively, and esiRNA against *Renilla* luciferase (MISSION esiRNA EHURLUC, Sigma-Aldrich) was used as control.

### Antibodies

The following primary and secondary antibodies were applied for immunodetection and immunostaining: anti-ataxin-3 (1:5000; 1H9, MAB5360, Merck, Darmstadt, Germany), anti-ataxin-3/C-terminal (1:2500; SA3637) [20], anti-KPNB1 (1:5000; H-300, sc-11367, Santa Cruz Biotechnology, Heidelberg, Germany), anti-KPNA3 (1:5000; ab137446, Abcam, Cambridge, United Kingdom), anti-CLPP (1:2500; 15,698–1-AP, Proteintech, St. Leon-Rot, Germany), anti-GAPDH (1:5000; 0411, sc-47724, Santa Cruz Biotechnology), anti-β-actin (1:5000; AC-15, A5441, Sigma-Aldrich), anti-vinculin (1:1000; E1E9V, 13,901, Cell Signaling Technologies, Frankfurt am Main, Germany), anti-lamin A/C (1:5000; 346, sc-7293, Santa Cruz Biotechnology), anti-GST (1:2500; B-14, sc-138, Santa Cruz Biotechnology), anti-α-spectrin (1:1000; AA6, Merck), anti-GFP (1:1000; B-2, sc-9996, Santa Cruz Biotechnology), anti-LC3 (1:1000; MBL-PD014, MBL International, Woburn, MA, US), anti-p62 (1:1000; 5114, Cell Signaling Technologies), and IRDye secondary antibodies goat anti-mouse 680LT, goat anti-mouse 800CW, goat anti-rabbit 680RD, goat anti-rabbit 800CW (all 1:10,000; LI-COR Biosciences, Bad Homburg, Germany), and Alexa Fluor 555 (1:500; Thermo Fisher Scientific).

### KPNB1 I178D cloning

Mutation on the nucleoporin FxFG binding site of KPNB1 leads to the reduction of both binding and nuclear transport of this protein [31]. I178D mutation was introduced by PCR. Briefly, the mutation (AAT to AGA) was amplified using the following primers: forward, 5’-atgtcacaaaccccaacagc-3′; and reverse, 5’-gtagcagctagcttcacattattactaggctcttctttccttcatcccctggTCTatggcagtcagaatc-3’, and introduced into the pCMV6-XL5 vector containing full-length human KPNB1 using SalI-HF and NheI restriction enzymes (New England Biolabs, Frankfurt am Main, Germany). Afterwards, it was subjected to Sanger sequencing using primers: forward, 5’-accagtggccagaactcatt-3’; and reverse, 5’-agtgcctttcagactctttatca-3’ to confirm the respective mutation and to ensure that no other mutations have been generated.

### Cell culture and transfection

Wild-type HEK 293T cells (ATCC: CRL-11268) and *ATXN3* KO HEK 293T cells previously generated in our lab [32], were maintained in Dulbecco’s modified Eagle’s medium (DMEM) supplemented with 10% fetal bovine serum (FBS) and 1% antibiotic–antimycotic (all Gibco, Thermo Fisher Scientific) in a mycoplasma-free incubator at 37 °C and 5% CO_2_. Transient transfection of HEK 293T cells with constructs and esiRNAs was carried out using TurboFectin 8.0 (OriGene Technologies) and Attractene (QIAGEN) transfection reagents, respectively, according to the manufacturer’s instructions.

Fibroblasts from three MJD patients and three healthy individuals were reprogrammed to iPSCs as described previously [33].

### Cell treatments

For KPNB1 inhibition, transfected cells were incubated with 16 µM importazole (IPZ; Sigma-Aldrich) for 48 h. To turn off the expression of Tet-off system, 4.5 µM doxycycline (Sigma-Aldrich) was applied. Activation of endogenous calpains was performed as previously described [18]. Briefly, calpains were activated by incubating transfected cells with 1 µM ionomycin (Sigma-Aldrich) and 5 mM CaCl_2_ for 1 h. To block major proteolytic pathways, transfected cells were treated with 10 µM calpain inhibitor III (CI-III; Sigma-Aldrich), 10 µM broad-spectrum caspase inhibitor Q-VD-OPh (Sigma-Aldrich), 50 nM autophagy inhibitor bafilomycin A1 (InvivoGen, Toulouse, France), or 10 µM proteasomal inhibitor lactacystin (Enzo Life Sciences, Lausen, Switzerland) for 18 h prior to harvesting.

### Cell viability assay

Cell viability was evaluated using the resazurin-based PrestoBlue assay (Thermo Fisher Scientific) according to the manufacturer’s instructions. In brief, 5000 *ATXN3* KO HEK 293T cells per well were seeded in a ViewPlate-96 Black (PerkinElmer, Waltham, MA, US) and transfected on the next day. After 72 h, culture medium was aspirated and 100 µl fresh medium containing PrestoBlue reagent in a ratio of 1:10 was added. Cells were incubated at 37 °C and 5% CO_2_ for 45 min. Afterwards, fluorescence signals were measured at 535 nm (excitation)/620 nm (emission) using an EnVision Multimode Plate Reader and the EnVision Manager software version 1.13.3009.1401 (both PerkinElmer).

### Protein lysate preparation

To prepare cell lysates, cells were harvested by removing the media, dissociating the cell layer by pipetting using cold Dulbecco’s phosphate-buffered saline (DPBS; Gibco, Thermo Fisher Scientific), and centrifugation at 300x*g* for 5 min. RIPA buffer (50 mM Tris pH 7.5, 150 mM NaCl, 1% Triton X-100, 0.1% SDS, 0.5% sodium deoxycholate) containing cOmplete protease inhibitor EDTA-free (Hoffmann-La Roche, Basel, Switzerland) was added to the cell pellets, incubated on ice for 25 min (vortexing every 5 min), and centrifuged at 16,000x*g* for 15 min at 4 °C. In the final step, the supernatant was transferred to a fresh reaction tube, supplemented with 10% glycerol, and stored at − 80 °C until further analysis. To obtain mouse brain lysates, brain hemispheres were homogenized in RIPA buffer containing cOmplete protease inhibitor EDTA-free using the ULTRA-TURRAX disperser (VWR International, Ulm, Germany). Subsequently, homogenates were centrifuged at 16,000x*g* for 30 min at 4 °C and followed as described. Protein concentrations of cell and mouse brain lysates were determined using Bradford protein assay (Bio-Rad Laboratories, Hercules, CA, US).

### Western blot analysis

30 μg protein extract were mixed with 4 x LDS sample buffer (1 M Tris-base pH 8.5, 2 mM EDTA, 8% lithium dodecyl sulfate (LDS), 40% glycerol, 0.025% phenol red) supplemented with 100 mM dithiothreitol (DTT) and heat-denatured for 10 min at 70 °C. Protein samples were subjected to electrophoretic separation using 10% Bis–Tris gels and MES SDS (50 mM MES, 50 mM Tris-base pH 7.3, 0.1% SDS, 1 mM EDTA) or MOPS SDS (50 mM MOPS, 50 mM Tris-Base pH 7.7, 0.1% SDS, 1 mM EDTA) running buffer. Afterwards, proteins were transferred onto Amersham Biosciences Protran Premium 0.2 µm nitrocellulose membranes (GE Healthcare) using Bicine/Bis–Tris transfer buffer (25 mM Bicine, 25 mM Bis–Tris pH 7.2, 1 mM EDTA) containing 15% methanol at 80 V for 90 min. After transfer, membranes were blocked using 5% skim milk (Sigma-Aldrich) in Tris-buffered saline (TBS; 10 mM Tris pH 7.5, 150 mM NaCl) for 1 h at room temperature and incubated with primary antibody diluted in TBS-T (TBS containing 0.1% Tween 20) overnight at 4 °C. For visualization, fluorescence-conjugated secondary antibodies were added to the membranes for 1 h at room temperature. Fluorescence signals were detected using the LI-COR ODYSSEY FC system (LI-COR Biosciences) and a ratio of target protein to loading control was quantified using Image Studio 5.2 software (LI-COR Biosciences).

### Filter retardation assay

Formation of SDS-insoluble aggregates was analyzed by filter retardation assay as described before [18]. *ATXN3* KO HEK 293T cells were harvested 72 h post-transfection and homogenized in lysis buffer (DPBS supplemented with 1% Triton X-100 and cOmplete protease inhibitor EDTA-free) by ultra-sonication for 10 s. Protein concentration of cell homogenates was measured using Bradford protein assay. 10 µg protein extract were diluted in total volume of 100 µl DPBS supplemented with 2% SDS and 50 mM DTT. Samples were boiled for 5 min at 95 °C and cooled down at room temperature to avoid precipitation of SDS. Afterwards, samples were filtered through Amersham Biosciences Protran 0.45 µm nitrocellulose membranes (GE Healthcare) using a Minifold II Slot Blot System (GE Healthcare). Membranes were blocked using 5% skim milk in TBS, and immunodetection was conducted as described in the western blot analysis.

### Nucleocytoplasmic fractionation assay

To investigate the subcellular localization of ataxin-3, cytoplasmic and nuclear fractions were separated according to the Rapid, Efficient And Practical (REAP) fractionation method [34] with minor modifications [18]. *ATXN3* KO HEK 293T cells were harvested 72 h post-transfection using DPBS and centrifuged at 300x*g* for 5 min. Cell pellets were triturated in lysis buffer (DPBS supplemented with 0.1% NP-40 and cOmplete protease inhibitor EDTA-free), and an aliquot of cell suspension was taken as the whole cell lysate. The residual volume was centrifuged at 10,000x*g* for 10 s and the supernatant was collected as the cytoplasmic fraction. Whole cell lysate and cytoplasmic fraction were mixed with 4 x LDS sample buffer and 100 mM DTT. The pellet was rinsed once with lysis buffer and resuspended in 1 x LDS sample buffer and 100 mM DTT, representing the nuclear fraction. All samples were heat-denatured for 10 min at 70 °C followed by ultra-sonication for 10 s. Ultimately, samples were subjected to western blot analysis and immunodetection according to the described protocol.

### GST pull-down assay

*E. coli* BL21 bacteria containing pGEX-6P-1-MJD15Q and pGEX-6P-1-MJD77Q were cultured overnight at 37 °C, and protein expression was induced by adding 1 mM isopropyl β-D-1-thiogalactopyranoside (IPTG) for 2 h. Bacteria were centrifuged at 1500x*g* for 5 min. Pellets were rinsed once and resuspended in GST binding buffer (4.2 mM NaH_2_PO_4_, 2 mM KH_2_PO_4_, 140 mM NaCl, 10 mM KCl, 1% NP-40) containing cOmplete protease inhibitor EDTA-free. Bacterial lysates were prepared by ultra-sonication for 15 s followed by centrifugation at 16,000x*g* for 10 min at 4 °C. Eukaryotic protein extracts were obtained by harvesting HEK 293T cells using DPBS and centrifugation at 300x*g* for 5 min at 4 °C. Pellets were resuspended and lysed in GST binding buffer and centrifuged at 16,000x*g* for 15 min at 4 °C. Protein concentrations of HEK 293T cells and bacterial lysates were determined using Bradford protein assay. 500 µg prokaryotic protein extract containing GST-15Q or GST-77Q ataxin-3 were immobilized on MagneGST Glutathione Particles (Promega, Walldorf, Germany) for 1 h at 4 °C followed by four times washing with GST binding buffer at 4 °C. Then, MagneGST Glutathione Particles were incubated with 500 µg HEK 293T cells protein extract overnight at 4 °C and washed four times with GST binding buffer. Trapped proteins were eluted from the particles by boiling in 1 x LDS sample buffer containing 100 mM DTT for 5 min at 95 °C and analyzed by western blotting and immunodetection.

### Immunofluorescence staining

*ATXN3* KO HEK 293T cells were grown on poly-L-lysine-coated glass coverslips and transfected with respective constructs for 72 h. For cell fixation, medium was aspirated and incubation with 4% paraformaldehyde (PFA) in DPBS was performed for 15 min at room temperature. Cells were washed by DPBS briefly, blocked and permeabilized using DPBS (supplemented with 10% BSA and 0.5% Triton X-100) for 1 h at room temperature. Primary antibody diluted in DPBS supplemented with 1% BSA and 0.5% Triton X-100 was added to the fixed cells and incubated overnight at 4 °C. The cells were washed with cold DPBS four times and incubated with fluorescence-tagged secondary antibody (Alexa Fluor 555) for 1 h at room temperature. Subsequently, cells were washed with cold DPBS four times and mounted using VECTASHIELD Antifade Mounting Medium containing DAPI (Vector Laboratories, Peterborough, UK). Fluorescent images were taken with an Axioplan 2 Imaging System, AxioCam MRm (Carl Zeiss Microscopy, Jena, Germany), 400 × magnification, and AxioVision version 4.8 imaging software (Carl Zeiss Microscopy). 20 fields of vision with at least 10 GFP-positive cells were photographed per coverslip under blinded condition. The number of cells with aggregates was counted manually and calculated as percentage of total GFP-positive cells.

### Protein in-gel digestion

*ATXN3* KO HEK 293T cells were transfected for 72 h in three biological replicates. The whole cell lysates were purified with SDS-PAGE (Thermo Fisher Scientific), and the gel was stained using NOVEX Colloidal Blue Staining Kit (Thermo Fisher Scientific). Stained gel pieces were excised and in-gel digested using trypsin as described previously [35]. Tryptic peptides were desalted using C18-StageTips [36] and subjected to liquid chromatography and tandem mass spectrometry (LC–MS/MS) analysis.

### Mass spectrometry and data processing

LC–MS/MS analysis was conducted on an Easy nano-LC (Thermo Fisher Scientific) coupled to an QExactiveHF mass spectrometer (Thermo Fisher Scientific) as described before [37]. Peptides were eluted applying a 60-min segmented gradient with a flow rate of 200 nl/min. The 20 most intensive peaks were selected for fragmentation with HCD.

MS data from all three replicates were processed utilizing MaxQuant software suite version 1.6.7.0 [38]. Database search was carried out using the Andromeda search engine [39], which is integrated in MaxQuant. MS/MS spectra were searched against a target-decoy human UniProt database comprising of 96,817 protein entries and 245 commonly observed contaminants. Cysteine carbamidomethylation was considered as the static modification, while methionine oxidation and acetylation of N-terminal residues were set as variable modifications. Initial mass tolerance was set to 4.5 ppm and 0.5 Da for precursor and fragment ions, respectively. Peptide and modification site identifications were determined at a false discovery rate (FDR) of 0.01, interpreted by the target-decoy approach [40]. Label-free algorithm was enabled, as was the “match between runs” option for samples within one biological replicate [41]. Label-free quantification (LFQ) protein intensities from the MaxQuant data output were employed for relative protein quantification. Bioinformatic analysis (Student’s *t*-test) was performed using the Perseus software package version 1.6.2.3 [42]. A *p*-value ≤ 0.05 was considered statistically significant and SO was set to 0 (SO = 0). Volcano plots were generated with FDR ≤ 0.01 and randomization was set to 250. Activation and inhibition of cellular pathways upon KPNB1 overexpression was predicted using the Ingenuity Pathway Analysis (IPA; QIAGEN).

### Statistical analysis

Data are presented as mean ± SEM. Statistical analysis was conducted using GraphPad Prism 6.00 software (GraphPad Software Inc., La Jolla, CA, US). Statistical significance of data sets was evaluated using one sample *t*-test, two-tailed Student’s *t*-test, or one-way ANOVA with Tukey’s post-test. Significance was considered at *p*-value ≤ 0.05. Statistical outliers determined by ROUT test (Q = 1%) were excluded from analysis.

## Results

### Ataxin-3 interacts with karyopherins, but KPNB1 modulation does not affect its intracellular localization

**Fig. 1:**
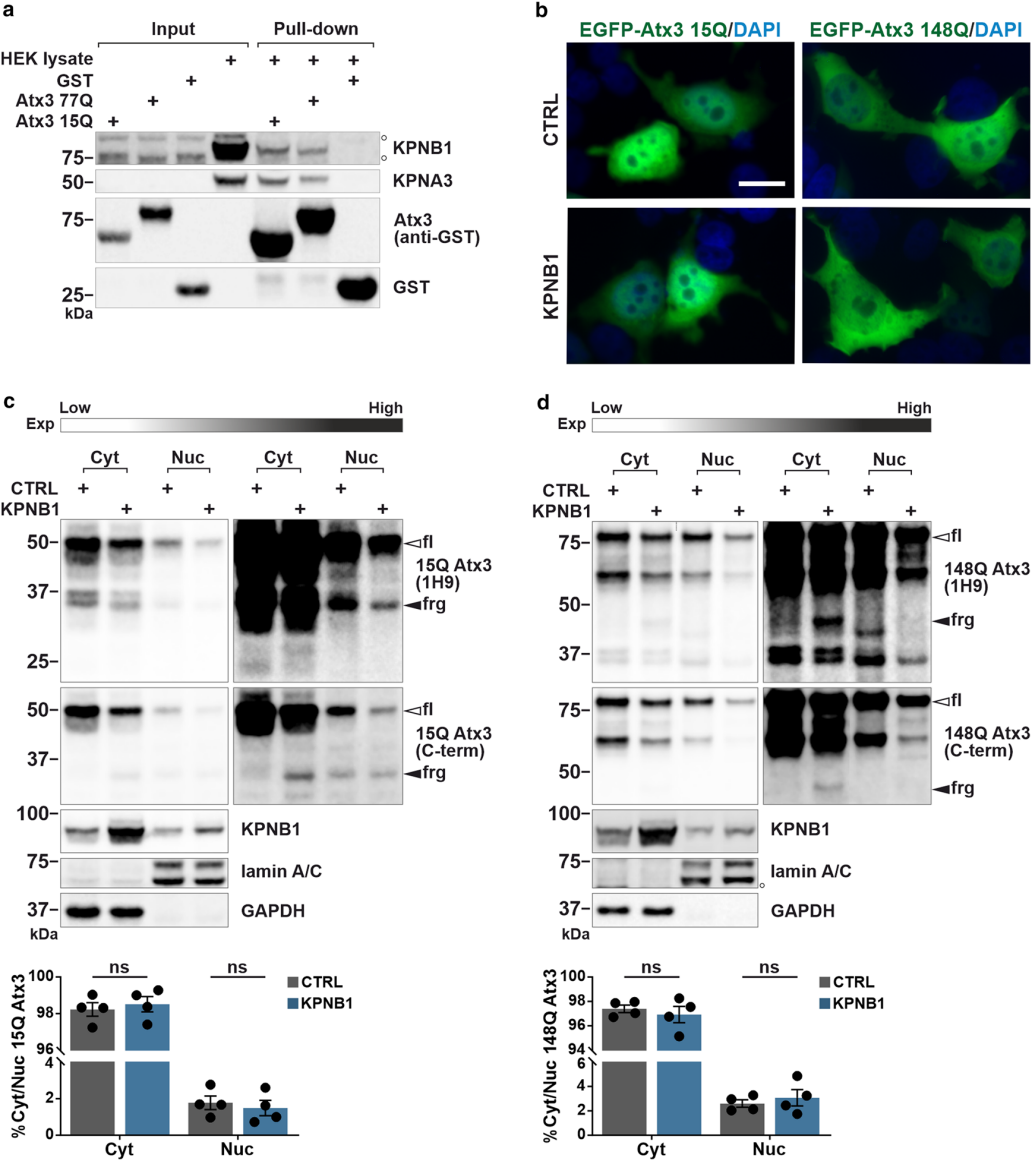
Ataxin-3 interacts with karyopherins, but KPNB1 overexpression does not affect the intracellular localization of ataxin-3. **a** Interaction of wild-type (15Q) and polyQ-expanded (77Q) ataxin-3 with KPNB1 and its partner KPNA3 was validated by GST pull-down assay. GST-tagged 15Q and 77Q ataxin-3 were purified using glutathione beads followed by incubation with wild-type HEK 293T cell lysates. Input as well as pull-down samples were assessed by western blot analysis. Empty GST was applied as negative control. White Bullets (o) indicate nonspecific bands. **b** Fluorescence microscopy was performed to visualize the subcellular localization of wild-type and polyQ-expanded ataxin-3 upon KPNB1 overexpression. *ATXN3* KO HEK 293T cells co-expressing either EGFP-ataxin-3 15Q or EGFP-ataxin-3 148Q and KPNB1 were analyzed 72 h post-transfection. Microscopy indicated that the intracellular localization of neither wild-type (15Q) nor polyQ-expanded (148Q) ataxin-3 was altered upon KPNB1 overexpression. Blue and green channels show DAPI as nuclear counterstain and GFP signals, respectively. 400 × magnification, scale bar = 20 µm. **c**, **d** Nucleocytoplasmic fractionation assay demonstrates a reduction of cytoplasmic as well as nuclear wild-type (15Q) and polyQ-expanded (148Q) ataxin-3 upon KPNB1 overexpression. However, no alteration was observed in the localization of ataxin-3. Moreover, accumulation of mainly cytoplasmic ataxin-3 fragments (black arrowheads) in KPNB1 overexpressing cells was observed. *ATXN3* KO HEK 293T cells were cotransfected with either 15Q or 148Q ataxin-3 and KPNB1 or empty vectors. 72 h post-transfection, nucleocytoplasmic fractionation was performed followed by western blot analysis. Blots are shown in low and high exposures. White bullet (o) marks nonspecific bands. Ataxin-3 was detected by 1H9 and C-terminal antibodies. The diagrams illustrate the ratio of nuclear and cytoplasmic ataxin-3 as percentage. GAPDH and lamin A/C were applied as cytoplasmic and nuclear loading controls, respectively. **c**, *n* = 4, Cyt/Nuc, unpaired *t*-test, *p* = 0.6261; **d**, *n* = 4, Cyt/Nuc, unpaired *t*-test, *p* = 0.5443. *CTRL* empty vector, *Cyt* cytoplasmic fraction, *Nuc* nuclear fraction, *fl* full-length, *frg* fragment, *C-term* C-terminal, *Exp* exposure. Values are displayed as means ± SEM. *ns* not significant

Nuclear shuttling of ataxin-3 is considered as a principal modulator of the MJD pathogenesis [22]. In our previous study, we have shown that KPNA3 has a leading role in the nucleocytoplasmic trafficking of ataxin-3, influencing its aggregation and toxicity [27]. Given that KPNA3 is an adaptor protein for KPNB1 to execute its transport function [24], we sought to study the impact of KPNB1 modulation on ataxin-3. In the first step, we tested whether ataxin-3 interacts directly with KPNB1 as well as KPNA3. For this, we performed a GST pull-down assay using GST-tagged wild-type (15Q) and polyQ-expanded (77Q) ataxin-3 overexpressed in *E. coli* and lysates of HEK 293T cells, which endogenously express KPNB1 and KPNA3. Western blot analysis of the GST pull-down showed that KPNB1, along with KPNA3, were coprecipitated with both 15Q and 77Q ataxin-3 (Fig. 1a), representing an important indication of a direct functional interaction between ataxin-3 and karyopherins. Next, we investigated the effect of KPNB1 overexpression on subcellular localization of ataxin-3 using fluorescence microscopy and nucleocytoplasmic fractionation. For this purpose, we transfected *ATXN3* KO HEK 293T cells with 15Q and 148Q ataxin-3 together with KPNB1 or an empty vector as a control. *ATXN3* KO HEK 293T cells were applied for most experiments to avoid the interference of endogenous ataxin-3, otherwise the cell type is mentioned. To our surprise, overexpression of KPNB1 did not show any significant alteration of the intracellular localization of ataxin-3 (Fig. 1b–d). However, KPNB1 overexpression led to an apparent reduction of both nuclear and cytoplasmic 15Q and 148Q ataxin-3 as observed in the western blot analysis of the nucleocytoplasmic fractionation assay (Figs. 1c, d, and S1a, b). Furthermore, the detections revealed the generation of specific ataxin-3 fragments, best visible in the cytoplasmic fraction (Fig. 1c, d). To assess the effect of KPNB1 knockdown on subcellular localization of ataxin-3, cells were cotransfected with either 15Q or 148Q ataxin-3 and endoribonuclease-prepared siRNA (esiRNA) targeting KPNB1 or *Renilla* luciferase (esiLUC) as a respective control. KPNB1 knockdown did not modulate the localization of ataxin-3, although in contrast to KPNB1 overexpression, an increase of both nuclear and cytoplasmic ataxin-3 was detected in these cells (Fig. S2a, b).

Overall, modulation of KPNB1 protein levels has a potential to influence ataxin-3 through a mechanism that does not appear to implicate the localization of this protein.

### KPNB1 overexpression lowers ataxin-3 protein levels independent of its nuclear transport function

**Fig. 2:**
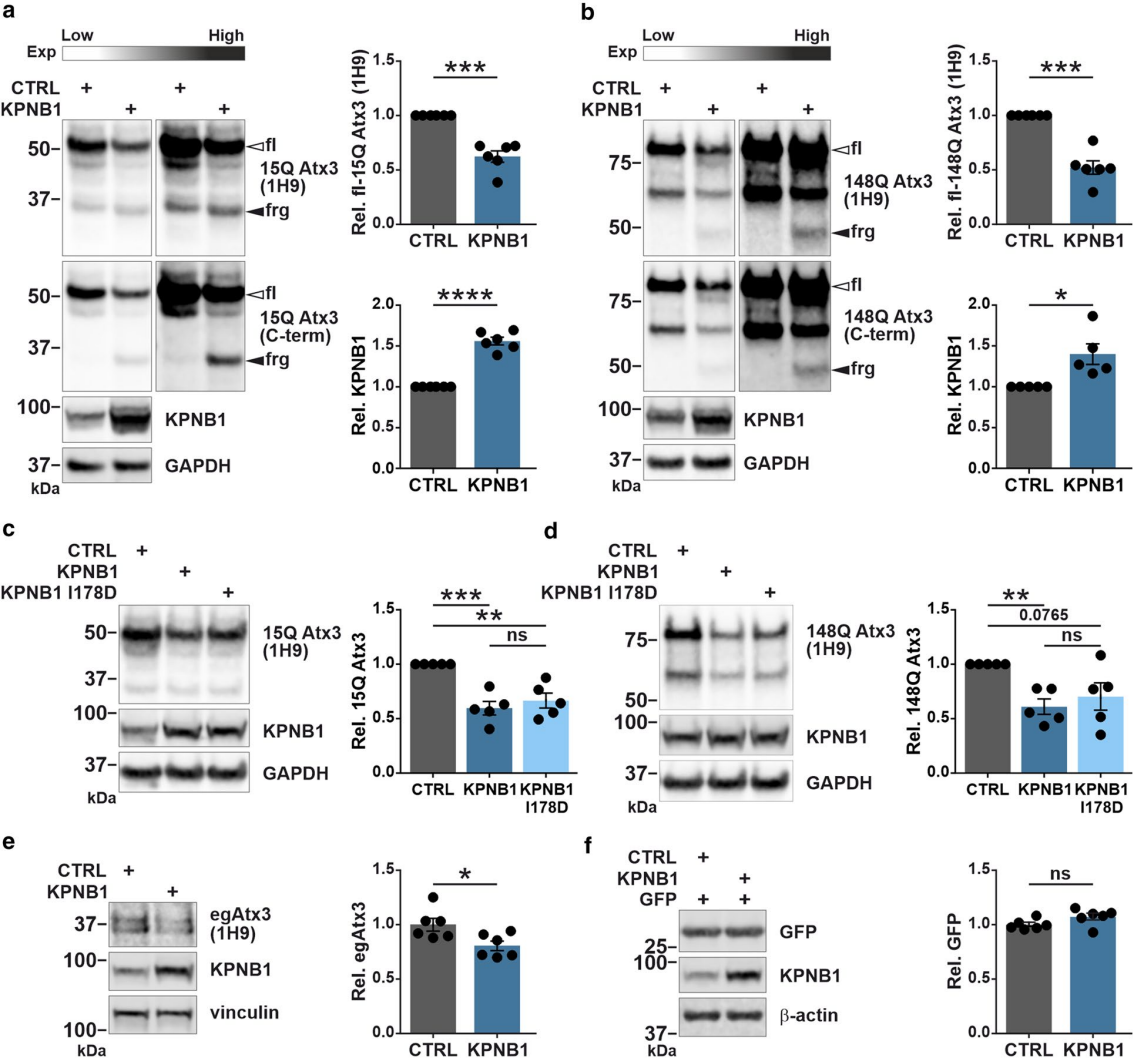
KPNB1 overexpression leads to a reduction of ataxin-3 protein levels, and mutation of the FxFG binding site in KPNB1 does not abolish this effect on ataxin-3. **a**,** b** KPNB1 overexpression decreases soluble levels of both wild-type (15Q) and polyQ-expanded (148Q) ataxin-3 and enhances fragmentation of ataxin-3 in comparison to control. *ATXN3* KO HEK 293T cells were cotransfected with either 15Q or 148Q ataxin-3 and KPNB1 or empty vectors for 72 h followed by western blot analysis. Detection of ataxin-3 using two different antibodies (1H9 and C-terminal) which recognize different epitopes revealed that ataxin-3 fragments are C-terminal breakdown products (black arrowheads). Western blot analysis confirmed the overexpression of KPNB1. Blots are shown in low and high exposures. GAPDH was applied as loading control. **a**, 15Q Atx3, *n* = 6, one sample *t*-test, *p* = 0.0008; KPNB1, *n* = 6, one sample *t*-test, *p* < 0.0001; **b**, 148Q Atx3, *n* = 6, one sample *t*-test, *p* = 0.0006; KPNB1, *n* = 5, one sample *t*-test, *p* = 0.0349. **c**, **d** KPNB1 mutation (I178D) in the FxFG binding site does not change the effect of KPNB1 overexpression on either wild-type or polyQ-expanded ataxin-3. *ATXN3* KO HEK 293T cells were cotransfected with either 15Q or 148Q ataxin-3 and wild-type KPNB1 or KPNB1 I178D constructs for 72 h. Western blot analysis displays that the ataxin-3-lowering effect of KPNB1 I178D is comparable to wild-type KPNB1, and it leads to a significant reduction of both 15Q and 148Q ataxin-3 protein levels. GAPDH was applied as loading control. **c**, *n* = 5, one sample *t*-test, CTRL *vs* KPNB1, *p* = 0.0030; CTRL *vs* KPNB1 I178D, *p* = 0.0086; unpaired *t*-test, KPNB1 *vs* KPNB1 I178D, *p* = 0.4784; **d**, *n* = 5, one sample *t*-test, CTRL *vs* KPNB1, *p* = 0.0052; CTRL *vs* KPNB1 I178D, *p* = 0.0765; unpaired *t*-test, KPNB1 *vs* KPNB1 I178D, *p* = 0.5367. **e** Western blot analysis of wild-type HEK 293T cells transfected with either KPNB1 or empty vectors. The result displays that KPNB1 overexpression is accompanied by a reduction in protein levels of endogenous ataxin-3. Vinculin was applied as loading control. *n* = 6, unpaired *t*-test, *p* = 0.0221. **f** Western blot analysis demonstrates that protein levels of GFP do not change upon KPNB1 overexpression. Wild-type HEK 293T cells were cotransfected with GFP and KPNB1 constructs for 72 h. β-actin was applied as loading control. *n* = 6, unpaired *t*-test, *p* = 0.0756. *CTRL* empty vector, *fl* full-length, *frg* fragment, *C-term* C-terminal, *Exp* exposure, *egAtx3* endogenous ataxin-3, *Rel.* relative. Values are displayed as means ± SEM. *ns* not significant; *p ≤ 0.05; **p ≤ 0.01; ***p ≤ 0.001; ****p ≤ 0.0001

After observing potential effects of KPNB1 overexpression on ataxin-3 protein levels in our nucleocytoplasmic fractionation assays, we sought to investigate in detail whether increasing the levels of KPNB1 has any robust effects on ataxin-3 protein levels. First, we transiently transfected cells with either 15Q or 148Q ataxin-3 and KPNB1 constructs. Western blot analysis revealed that overexpression of KPNB1 was accompanied by a significant reduction in the total amount of both 15Q and 148Q ataxin-3 (Fig. 2a, b). In addition to decreased protein levels of ataxin-3, we confirmed an enhanced fragmentation of ataxin-3 upon KPNB1 overexpression. Using two different antibodies recognizing different epitopes, we concluded that these fragments are C-terminal and containing the polyQ tract (Fig. 2a, b). Further analysis demonstrated that the accumulation of ataxin-3 fragments as well as the reduction of full-length ataxin-3 was time-dependent, with the strongest effect being observed after 72 h of KPNB1 overexpression (Fig. S3a, b).

Nuclear translocation of cargos mediated by transport receptors involves the interaction of karyopherin beta with nuclear pore complex proteins (nucleoporins) containing phenylalanine-rich core motifs (FxFG) [43]. It was shown that mutation of isoleucine 178 (I178) in the FxFG binding site of KPNB1 to the less hydrophobic aspartic acid (D), remarkably hampers both binding and nuclear transport of KPNB1 [31]. KPNB1 I178D as well as wild-type KPNB1 were overexpressed in cells transiently expressing either 15Q or 148Q ataxin-3. The effects on full-length ataxin-3 upon KPNB1 I178D overexpression were dissected by western blot analysis. Interestingly, no major difference was identified between the impact of KPNB1 I178D and wild-type KPNB1 on soluble ataxin-3 (Fig. 2c, d), implying that the observed ataxin-3 lowering is a nuclear transport function-independent mechanism.

Next, we wanted to confirm whether KPNB1 overexpression affects the protein levels of endogenous ataxin-3. Moreover, to determine if the impact of KPNB1 on ataxin-3 is substrate-specific, we analyzed the effect of KPNB1 overexpression on a control protein such as GFP. According to its effects on overexpressed ataxin-3, western blot analysis displayed a significant decrease in the protein levels of endogenous ataxin-3 upon KPNB1 overexpression (Fig. 2e), whereas no alteration was observed in the levels of GFP (Fig. 2f). The above results highlight that ataxin-3 protein levels are generally yet specifically lowered by KPNB1 overexpression. These alterations yield KPNB1 as a putative modulator of ataxin-3 protein levels independent of the KPNB1 nuclear transport function.

### Knockdown and pharmacological inhibition of KPNB1 increase ataxin-3 protein levels

**Fig. 3:**
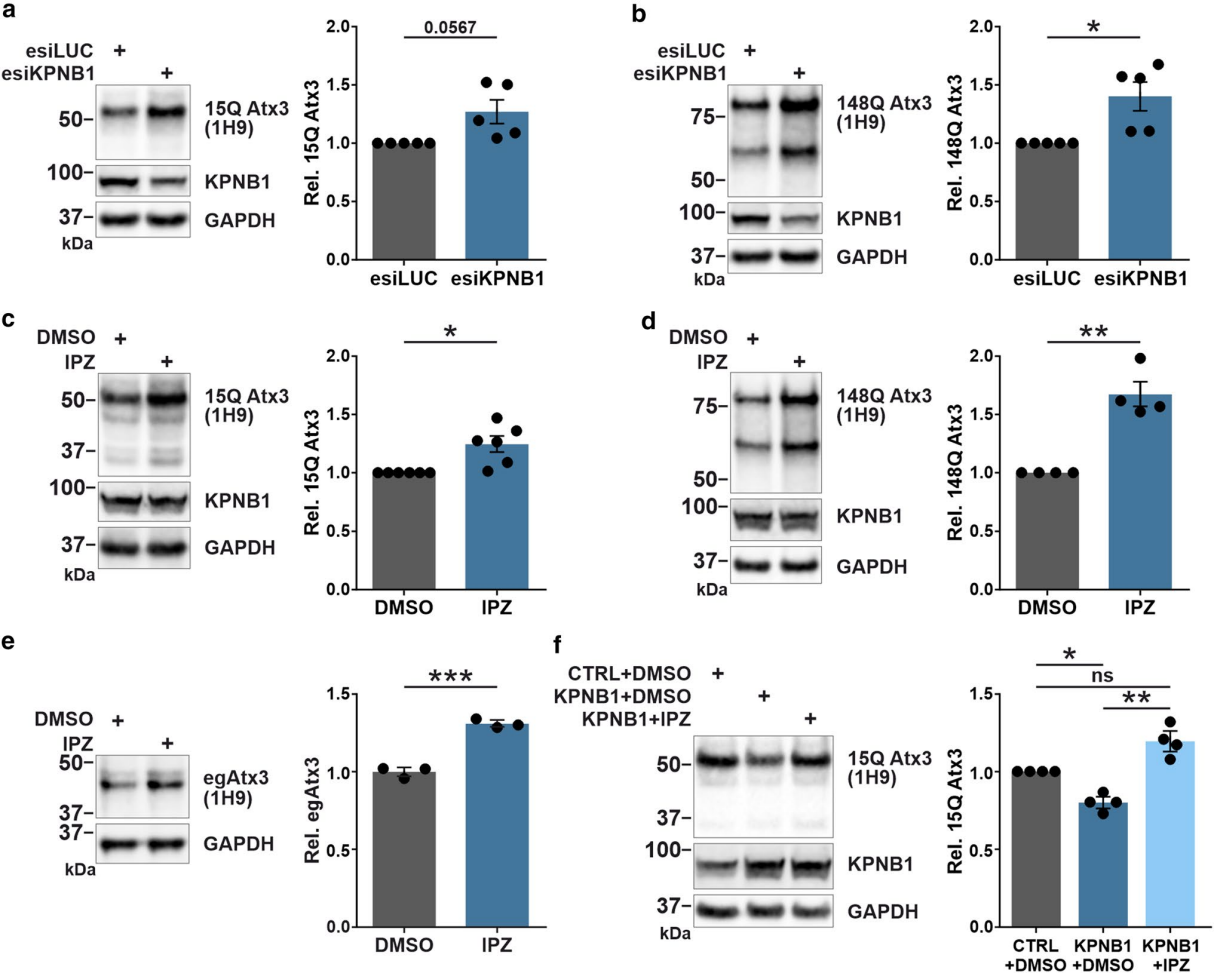
Knockdown and pharmacological inhibition of KPNB1 elevate ataxin-3 protein levels. **a**, **b** Western blot analysis reveals that knockdown of KPNB1 is accompanied by a significant increase in the soluble levels of wild-type (15Q) and polyQ-expanded (148Q) ataxin-3. *ATXN3* KO HEK 293T cells were cotransfected with either 15Q or 148Q ataxin-3 and esiKPNB1 or esiLUC (control). Cells were harvested 72 h post-transfection, and cell lysates were subjected to western blotting. GAPDH was applied as loading control. **a**, *n* = 5, one sample *t*-test, *p* = 0.0567; **b**, *n* = 5, one sample *t*-test, *p* = 0.0315. **c**, **d** KPNB1 was inhibited using 16 µM importazole (IPZ) in either wild-type (15Q) or polyQ-expanded (148Q) ataxin-3 expressing HEK 293T cells. 48 h after treatment, cells were harvested and subjected to western blot analysis. Quantification of blots demonstrates that the soluble levels of both 15Q and 148Q ataxin-3 increase upon IPZ treatment compared with DMSO-treated cells (control). GAPDH was applied as loading control. **c**, *n* = 6, one sample *t*-test, *p* = 0.0162; **d**, *n* = 4, one sample *t*-test, *p* = 0.0077. **e** Western blot analysis shows a significant increase of endogenous ataxin-3 protein levels upon IPZ treatment compared with control. Wild-type HEK 293T cells were treated with either 16 µM IPZ or DMSO for 48 h prior to harvesting. GAPDH was applied as loading control. *n* = 3, unpaired *t*-test, *p* = 0.0009. **f** Protein levels of ataxin-3 can be rescued by IPZ treatment in KPNB1 overexpressing cells. *ATXN3* KO HEK 293T cells were cotransfected with 15Q ataxin-3 and KPNB1 constructs followed by 16 µM IPZ or DMSO treatment for 48 h prior to harvesting. Cells were harvested 72 h post-transfection, and ataxin-3 levels were analyzed by western blotting. GAPDH was applied as loading control. *n* = 4, one sample *t*-test, CTRL + DMSO *vs* KPNB1 + DMSO, *p* = 0.0129; CTRL + DMSO *vs* KPNB1 + IPZ, *p* = 0.0615; unpaired *t*-test, KPNB1 + DMSO *vs* KPNB1 + IPZ, *p* = 0.0021. *CTRL* empty vector, *IPZ* importazole, *egAtx3* endogenous ataxin-3, *Rel.* relative. Values are displayed as means ± SEM. *ns*  not significant; *p ≤ 0.05; **p ≤ 0.01; ***p ≤ 0.001

In the next step, we explored the consequences of lowering KPNB1 levels on wild-type and polyQ-expanded ataxin-3. Cells were transiently transfected with either 15Q or 148Q ataxin-3 together with esiKPNB1 or esiLUC as a respective control and analyzed by western blotting. In contrast to overexpression of KPNB1, knockdown of this protein resulted in an increase in soluble levels of both full-length 15Q and 148Q ataxin-3 (Figs. 3a, b, and S4).

To further validate our findings, we targeted KPNB1 by pharmacological inhibition. For this, we employed 2,4-diaminoquinazoline (importazole/IPZ) as a specific and reversible inhibitor of KPNB1. The mode of action of IPZ is likely via altering the interaction of KPNB1 with RanGTP [44]. *ATXN3* KO HEK 293T cells transiently expressing 15Q or 148Q ataxin-3 as well as wild-type HEK 293T cells were treated with IPZ, and ataxin-3 protein levels were assessed by western blotting. Treatment with IPZ recapitulated the effects of KPNB1 knockdown on ataxin-3 protein levels, demonstrating significantly elevated levels of soluble endogenous or overexpressed 15Q and 148Q ataxin-3 (Fig. 3c–e). To additionally scrutinize the decrease of ataxin-3 protein levels by KPNB1, cells overexpressing 15Q ataxin-3 together with KPNB1 were treated with IPZ. Interestingly, the reduction of ataxin-3 protein levels upon KPNB1 overexpression was restored via IPZ treatment (Fig. 3f). Altogether, both KPNB1 knockdown and its pharmacological inhibition using IPZ led to an increase in soluble levels of wild-type and polyQ-expanded ataxin-3, while the overexpression of KPNB1 had an opposite effect.

### KPNB1 overexpression reduces ataxin-3 stability and gives rise to fragments independent of MJD-associated proteolytic pathways

**Fig. 4:**
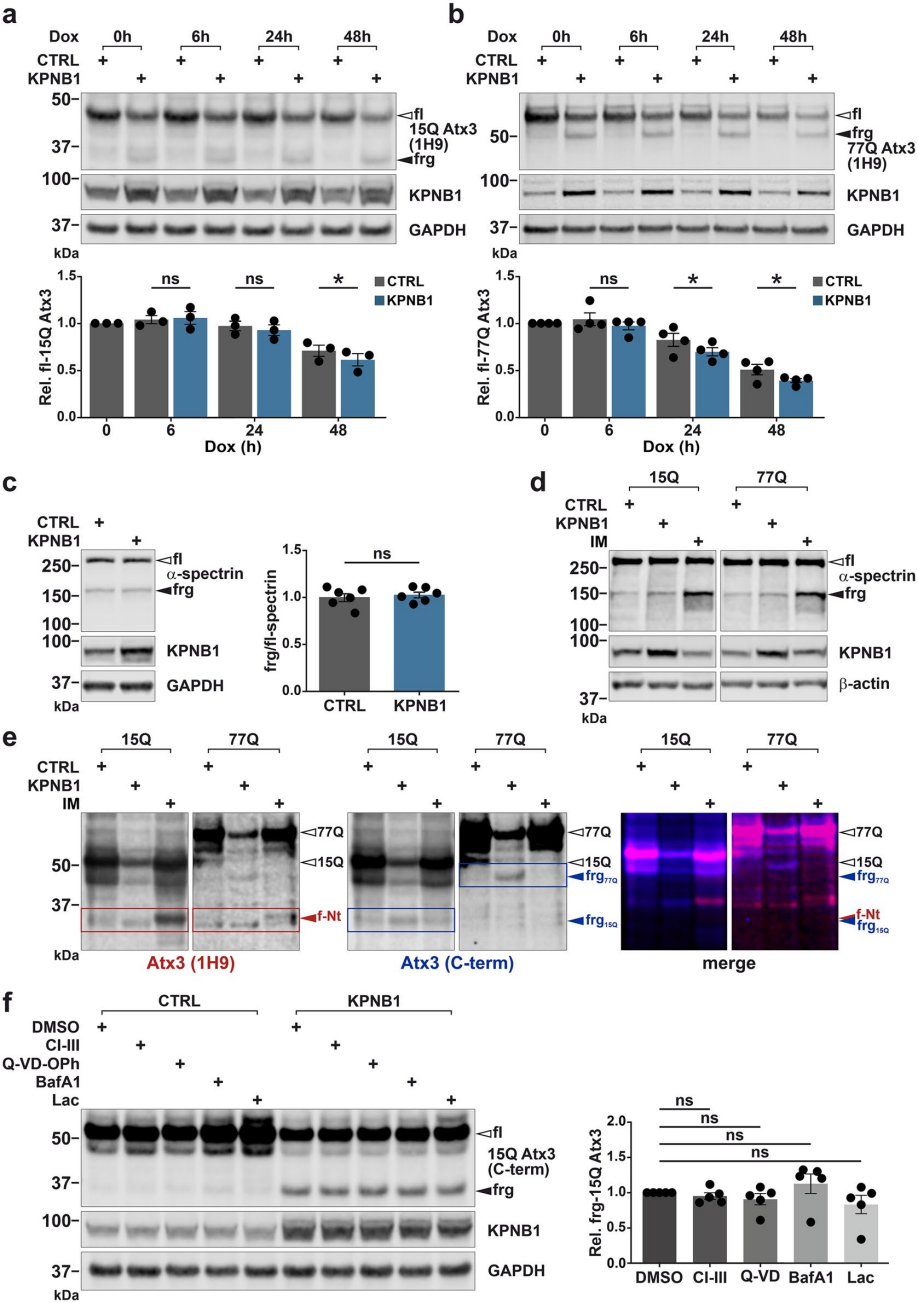
KPNB1 overexpression decreases the stability of ataxin-3, and observed ataxin-3 fragments are not mediated by the activation of known MJD-associated proteases or proteolytic pathways. **a**, **b** Modulation of ataxin-3 stability was analyzed upon KPNB1 overexpression using the Tet-off system. Western blot analysis revealed a significant reduction in the stability of both wild-type (15Q) and polyQ-expanded (77Q) ataxin-3 in KPNB1 overexpressing cells 48 h after termination of ataxin-3 expression compared with control. *ATXN3* KO HEK 293T cells were cotransfected with either pTRE-15Q or pTRE-77Q ataxin-3 and KPNB1 or empty vectors. 24 h post-transfection, ataxin-3 expression was shut off using doxycycline (Dox) at different time intervals (0 h, 6 h, 24 h, and 48 h). GAPDH was applied as loading control. **a**, *n* = 3, paired *t*-test, CTRL *vs* KPNB1 (6 h), *p* = 0.6029; CTRL *vs* KPNB1 (24 h), *p* = 0.6730; CTRL *vs* KPNB1 (48 h), *p* = 0.0132; **b**, *n* = 4, paired *t*-test, CTRL *vs* KPNB1 (6 h), *p* = 0.3859; CTRL *vs* KPNB1 (24 h), *p* = 0.0202; CTRL *vs* KPNB1 (48 h), *p* = 0.0427. **c** Activation of calpains and caspases upon KPNB1 overexpression was evaluated by detecting cleavage of their common substrate α-spectrin protein in wild-type HEK 293T cells. Western blot analysis demonstrates no alteration in the levels of either full-length (white arrowhead) or breakdown products of α-spectrin (black arrowhead). Moreover, ratio between cleaved and full-length protein remained comparable to control in KPNB1 overexpressing cells. GAPDH was applied as loading control. *n* = 6, unpaired *t*-test, *p* = 0.6296. **d** Breakdown products of α-spectrin (black arrowhead) are induced in ionomycin (IM) treated cells confirming the activation of calpains compared with control. *ATXN3* KO HEK 293T cells were cotransfected with either 15Q or 77Q ataxin-3 followed by KPNB1 overexpression or 1 µM IM treatment for 1 h. β-actin was applied as loading control. **e** IM administration results in the activation of endogenous calpains and cleavage of ataxin-3. Western blot analysis displays that ataxin-3 fragments induced by KPNB1 overexpression (blue boxes and arrowheads) are not comparable to fragments mediated by calpain cleavage (red boxes and arrowheads). Red and blue channels indicate ataxin-3 detected by 1H9 and C-terminal antibodies, respectively. **f** Western blot analysis demonstrates that inhibition of calpains and caspases or blocking of autophagy and proteasomal degradation do not prevent ataxin-3 fragmentation in KPNB1 overexpressing cells. *ATXN3* KO HEK 293T cells cotransfected with 15Q ataxin-3 and KPNB1 or empty vectors were incubated with 10 µM calpain inhibitor III (CI-III), 10 µM caspase inhibitor (Q-VD-OPh), 50 nM autophagy inhibitor bafilomycin A1 (BafA1), or 10 µM proteasomal inhibitor lactacystin (Lac) for 16 h prior to harvesting. Cells incubated with DMSO were considered as control. The diagram illustrates quantification of ataxin-3 fragments in KPNB1 overexpressing cells. GAPDH was applied as loading control. *n* = 5, one sample *t*-test, DMSO *vs* CI-III, *p* = 0.3708; DMSO vs Q-VD, *p* = 0.3136; DMSO vs BafA1, *p* = 0.4063; DMSO vs Lac, *p* = 0.2682. *CTRL* empty vector, *fl* full-length, *frg* fragment, *Dox* Doxycycline, *C-term* C-terminal, *f-Nt* fragment-N-terminal, *IM* ionomycin, *CI-III* calpain inhibitor III, *BafA1* bafilomycin A1, *Lac* lactacystin, *Rel.* relative. Values are displayed as means ± SEM. *ns* not significant; *p ≤ 0.05

To unravel whether KPNB1 overexpression decreases ataxin-3 stability, we applied the Tet-off system for abrogating the expression of 15Q and 77Q ataxin-3 by administering doxycycline in a time-dependent manner. Western blot analysis indicated that the degradation of both wild-type and polyQ-expanded ataxin-3 accelerated significantly in KPNB1 overexpressing cells as observed 48 h after termination of 15Q ataxin-3, and 24 h and 48 h after termination of 77Q ataxin-3 expression. However, no alteration was observed in the generation of ataxin-3 fragments as they remained stable during the course of the assay (Fig. 4a, b). This result motivated us to investigate the fragmentation of ataxin-3 in KPNB1 overexpressing cells more closely.

Proteolytic degradation and cleavage of polyQ proteins such as ataxin-3, carried out by caspases and calpains or proteolytic pathways such as autophagy and proteasomes, are involved in the pathogenesis of polyQ diseases [45–47]. Notably, the activation of caspases and calpains has been identified in numerous MJD models [17, 48]. Therefore, we postulated that the elevated fragmentation of ataxin-3 in KPNB1 overexpressing cells might be mediated by the activation of caspases or calpains. For this purpose, the cleavage of α-spectrin, a natural substrate of caspases and calpains [49] was evaluated in KPNB1 overexpressing wild-type HEK 293T cells. Western blot analysis displayed that neither full-length nor breakdown products of α-spectrin were modulated by KPNB1 overexpression, and the ratio between them was comparable to control (Fig. 4c). To compare the cleavage pattern of ataxin-3 followed by KPNB1 overexpression and calpain activation, a cell-based calpain activation assay was conducted using the ionophore ionomycin (IM). Cells transiently expressing 15Q or 77Q ataxin-3 were subjected to either KPNB1 overexpression or IM treatment to trigger the activation of endogenous calpains, which was confirmed by increased α-spectrin cleavage (Fig. 4d). Using two different antibodies recognizing distinct epitopes, we observed that the cleavage pattern of 15Q or 77Q ataxin-3 followed by activation of calpains differed from ataxin-3 fragments induced by KPNB1 overexpression (Fig. 4e). Moreover, inhibition of calpains and caspases or blocking of autophagic and proteasomal degradation did not prevent the formation of KPNB1 overexpression-associated fragments (Figs. 4f, and S5).

These results provide evidence that the enhancement of ataxin-3 lowering and fragmentation in KPNB1 overexpressing cells does not occur via the activation of known MJD-associated proteases or proteolytic pathways.

### KPNB1 overexpression diminishes ataxin-3 aggregation and rescues cell viability

**Fig. 5:**
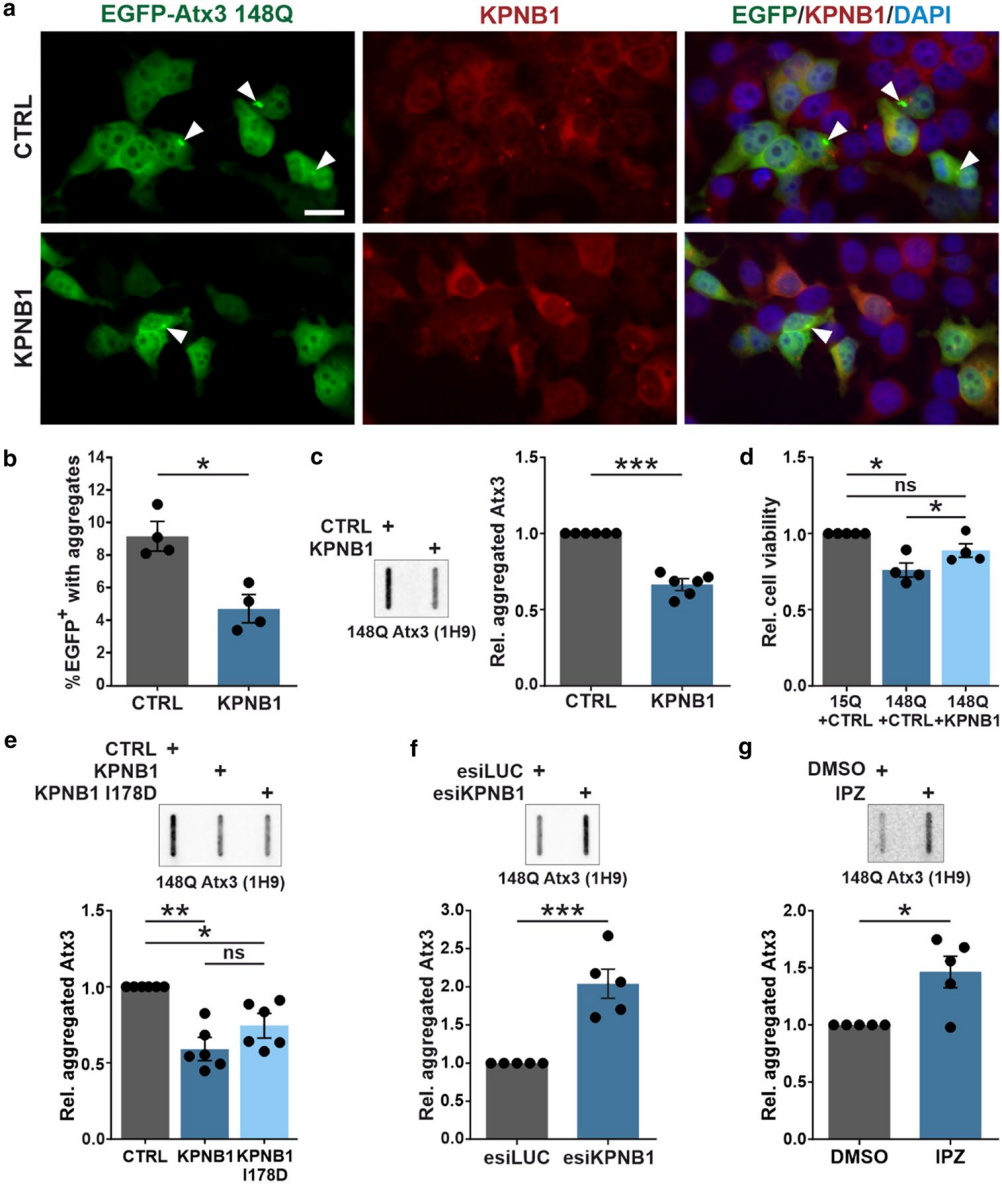
KPNB1 modulation affects ataxin-3 aggregation levels. **a**, **b** Fluorescence microscopy was conducted to visualize the alteration of polyQ-expanded ataxin-3 aggregation in KPNB1 overexpressing cells. *ATXN3* KO HEK 293T cells co-expressing EGFP ataxin-3 148Q and KPNB1 were fixed 72 h post-transfection, and the number of GFP-positive (EGFP^+^) cells with and without aggregates was counted manually in 20 fields of vision. The analysis showed a decrease of cells with ataxin-3 aggregates upon KPNB1 overexpression. Blue, green, and red channels show DAPI as nuclear counterstain, GFP, and KPNB1 signals, respectively. White arrowheads mark ataxin-3 aggregates. 400 × magnification, scale bar = 20 µm. The diagram shows the percentage of aggregates in EGFP^+^ cells. *n* = 4, unpaired *t*-test, *p* = 0.0123. **c** Filter retardation assay of *ATXN3* KO HEK 293T cells co-expressing polyQ-expanded (148Q) ataxin-3 and KPNB1 for 72 h demonstrated a reduction of polyQ-expanded ataxin-3 aggregates compared with control. *n* = 6, one sample *t*-test, *p* = 0.0004. **d** PrestoBlue assay of *ATXN3* KO HEK 293T cells cotransfected with either 15Q or 148Q ataxin-3 and KPNB1 or empty vectors. The viability of cells expressing 148Q ataxin-3 was rescued upon KPNB1 overexpression. Viability was normalized to 15Q ataxin-3 expressing cells. *n* = 4, one sample *t*-test, 15Q + CTRL *vs* 148Q + CTRL, *p* = 0.0144; 15Q + CTRL *vs* 148Q + KPNB1, *p* = 0.0882; paired *t*-test, 148Q + CTRL *vs* 148Q + KPNB1, *p* = 0.0138. **e** Filter retardation assay of *ATXN3* KO HEK 293T cells co-expressing 148Q ataxin-3 and either wild-type or KPNB1 I178D indicates a decrease in the aggregation of 148Q ataxin-3. *n* = 6, one sample *t*-test, CTRL *vs* KPNB1, *p* = 0.0030; CTRL *vs* KPNB1 I178D, *p* = 0.0247; unpaired *t*-test, KPNB1 *vs* KPNB1 I178D, *p* = 0.1883. **f** Knockdown of KPNB1 using esiRNA shows an increased load of aggregated 148Q ataxin-3 using filter retardation assay. *ATXN3* KO HEK 293T cells were cotransfected for 72 h with 148Q ataxin-3 and esiKPNB1 or esiLUC as control. *n* = 5, one sample *t*-test, *p* = 0.0054. **g** Filter retardation assay shows increased ataxin-3 aggregation in *ATXN3* KO HEK 293T cells expressing 148Q ataxin-3 for 72 h and treated with 16 µM IPZ for 48 h. Values were normalized to DMSO-treated cells. *n* = 5, one sample *t*-test, *p* = 0.0279. *CTRL* empty vector, *IPZ* importazole, *Rel.* relative. Values are displayed as means ± SEM. *ns*  not significant; *p ≤ 0.05; **p ≤ 0.01; ***p ≤ 0.001

Aggregation of polyQ-expanded ataxin-3 is a hallmark of MJD, and measuring the amount of protein aggregates can provide information on protein toxicity and disease progression [50]. As KPNB1 overexpression strongly reduced soluble levels of both wild-type and polyQ-expanded ataxin-3, we sought to investigate its consequences on the protein aggregation. Thus, aggregation of 148Q ataxin-3 upon KPNB1 overexpression was evaluated by fluorescence microscopy of cells transfected with EGFP-ataxin-3 148Q and KPNB1 constructs (Fig. 5a). In cells overexpressing KPNB1 the percentage of GFP-positive cells with aggregates reduced significantly compared with control (Fig. 5b). This finding was also confirmed by filter retardation assay, where a significant reduction of polyQ-expanded ataxin-3 aggregation was detected in KPNB1 overexpressing cells (Fig. 5c). Moreover, PrestoBlue assay revealed that KPNB1 overexpression rescues the impaired viability of 148Q ataxin-3 expressing cells compared with control (Fig. 5d). In line with the effects on soluble polyQ-expanded ataxin-3, overexpression of mutant KPNB1 (I178D) led to the reduction of ataxin-3 aggregates as well as overexpression of wild-type KPNB1 (Fig. 5e). Conversely, aggregation levels of 148Q ataxin-3 upon KPNB1 knockdown or inhibition with IPZ increased remarkably (Fig. 5f, g), corresponding to the increased soluble levels of polyQ-expanded ataxin-3.

### KPNB1 lowers ataxin-3 protein levels via activation of the mitochondrial protease CLPP

**Fig. 6:**
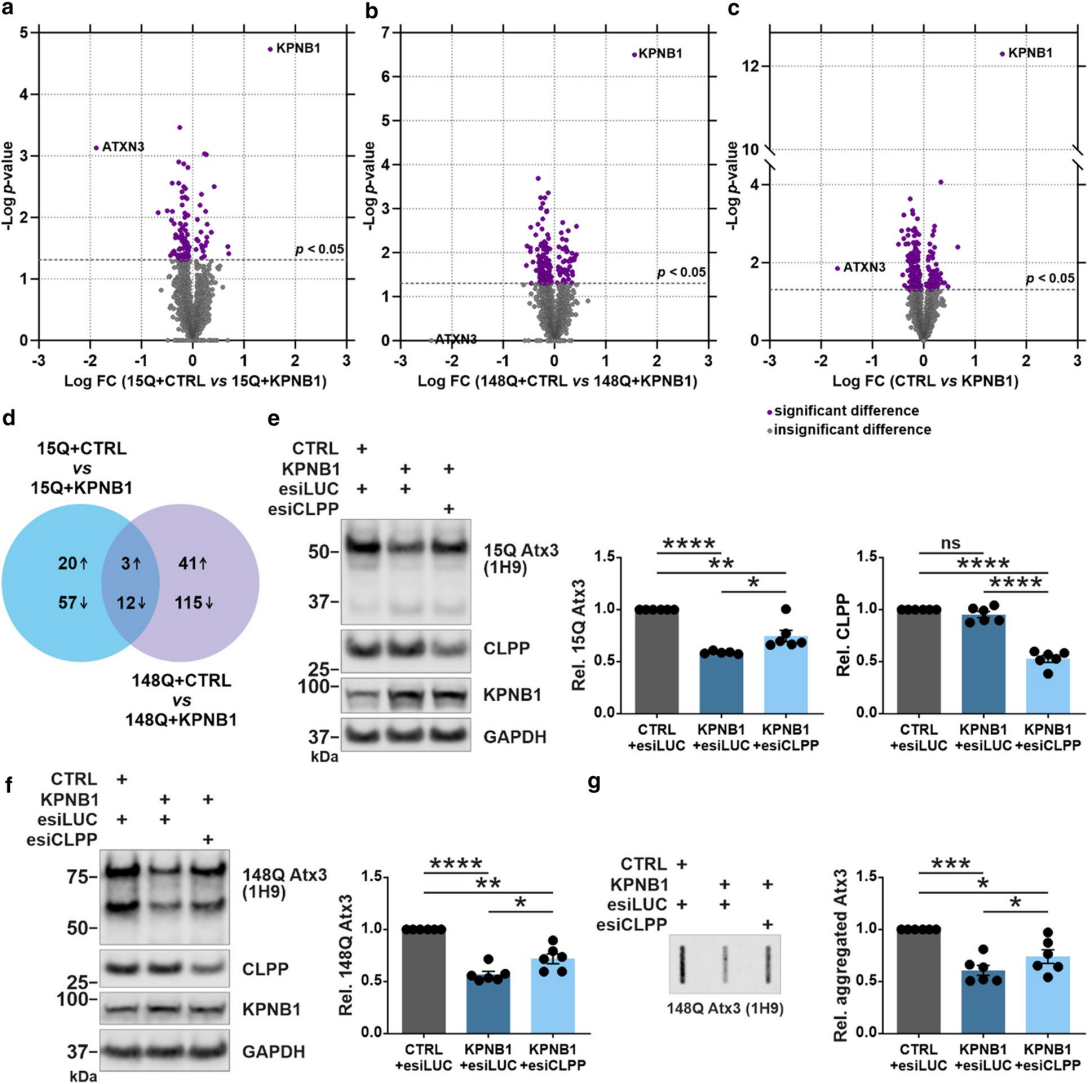
KPNB1 lowers ataxin-3 levels via activation of the mitochondrial protease CLPP. **a**–**c** Label-free quantitative proteomics of cells expressing ataxin-3 reveals activation of mitochondrial protease CLPP upon KPNB1 overexpression. Volcano plots indicating the upregulated and downregulated proteins in *ATXN3* KO HEK 293T cells cotransfected with either wild-type (15Q) or polyQ-expanded (148Q) ataxin-3 and KPNB1 or empty vectors. Volcano plots illustrate a direct comparison between the *p-*value and fold-change of the proteins. Purple circles show proteins with significantly altered levels upon KPNB1 overexpression. The thick dashed line represents the cut-off value at *p* = 0.05. *n* = 3, two-sample *t*-test, *p* ≤ 0.05. **d** Venn diagram displays the number of common and unique proteins which have been significantly upregulated or downregulated upon KPNB1 overexpression. **e**, **f** Western blot analysis demonstrates that both 15Q and 148Q ataxin-3 protein levels can be rescued partly by knockdown of CLPP in KPNB1 overexpressing cells. *ATXN3* KO HEK 293T cells were cotransfected with either 15Q or 148Q ataxin-3, KPNB1, and esiCLPP or esiLUC as control. Cells were harvested 72 h post-transfection and protein levels assessed by western blotting. GAPDH was applied as loading control. **e**, 15Q Atx3, *n* = 6, one sample *t*-test, CTRL + esiLUC *vs* KPNB1 + esiLUC, *p* < 0.0001; CTRL + esiLUC *vs* KPNB1 + esiCLPP, *p* = 0.0056; unpaired *t*-test, KPNB1 + esiLUC *vs* KPNB1 + esiCLPP,* p* = 0.0264; CLPP, *n* = 6, one sample *t*-test, CTRL + esiLUC *vs* KPNB1 + esiLUC, *p* = 0.1619; CTRL + esiLUC *vs* KPNB1 + esiCLPP, *p* < 0.0001; unpaired *t*-test, KPNB1 + esiLUC *vs* KPNB1 + esiCLPP, *p* < 0.0001; **f** *n* = 6, one sample *t*-test, CTRL + esiLUC *vs* KPNB1 + esiLUC, *p* < 0.0001; CTRL + esiLUC *vs* KPNB1 + esiCLPP, *p* = 0.0020; unpaired *t*-test, KPNB1 + esiLUC *vs* KPNB1 + esiCLPP, *p* = 0.0219. **g** Knockdown of CLPP in *ATXN3* KO HEK 293T cells cotransfected with 148Q ataxin-3 and KPNB1 for 72 h counteracts the KPNB1-induced lowering of polyQ-expanded ataxin-3 aggregates compared with control. *n* = 6, one sample *t*-test, CTRL + esiLUC *vs* KPNB1 + esiLUC, *p* = 0.0005; CTRL + esiLUC *vs* KPNB1 + esiCLPP, *p* = 0.0107; paired *t*-test, KPNB1 + esiLUC *vs* KPNB1 + esiCLPP,* p* = 0.0187. *CTRL* empty vector, *FC* fold-change, *Rel.* relative. Values are displayed as means ± SEM. *ns* not significant; *p ≤ 0.05; **p ≤ 0.01; ***p ≤ 0.001; ****p ≤ 0.0001

Given that KPNB1 overexpression leads to reduction of ataxin-3 protein levels and enhancement of ataxin-3 fragmentation, a question remains concerning which factor directly mediates this effect. To figure out the downstream pathways that might be implicated in this process, unbiased label-free quantitative proteomics was carried out using cells transiently overexpressing either 15Q or 148Q ataxin-3 and KPNB1 or empty vectors (control). Statistical analysis indicated that 23 proteins were upregulated, and 69 proteins downregulated significantly in cells overexpressing 15Q ataxin-3 and KPNB1 compared with control. In addition, 44 upregulated and 127 downregulated proteins were revealed in cells expressing 148Q ataxin-3 upon KPNB1 overexpression, and amongst them, 15 proteins (3 upregulated and 12 downregulated) were shared between 15Q and 148Q ataxin-3 expressing cells which are listed in Table S1 (Fig. 6a–d, and Table S1). To get insight into upstream regulators and identification of cellular processes that have been activated or inhibited, we applied the Ingenuity Pathway Analysis (IPA). Accordingly, activation of mitochondrial protease CLPP was predicted by IPA in both 15Q (*z*-score = 2, *p* = 1.43*e *− 04), and 148Q (*z*-score = 3.464, *p* = 7.52*e *− 14) ataxin-3 expressing cells upon KPNB1 cotransfection, as target proteins of CLPP (Table S2) were modulated.

**Fig. 7:**
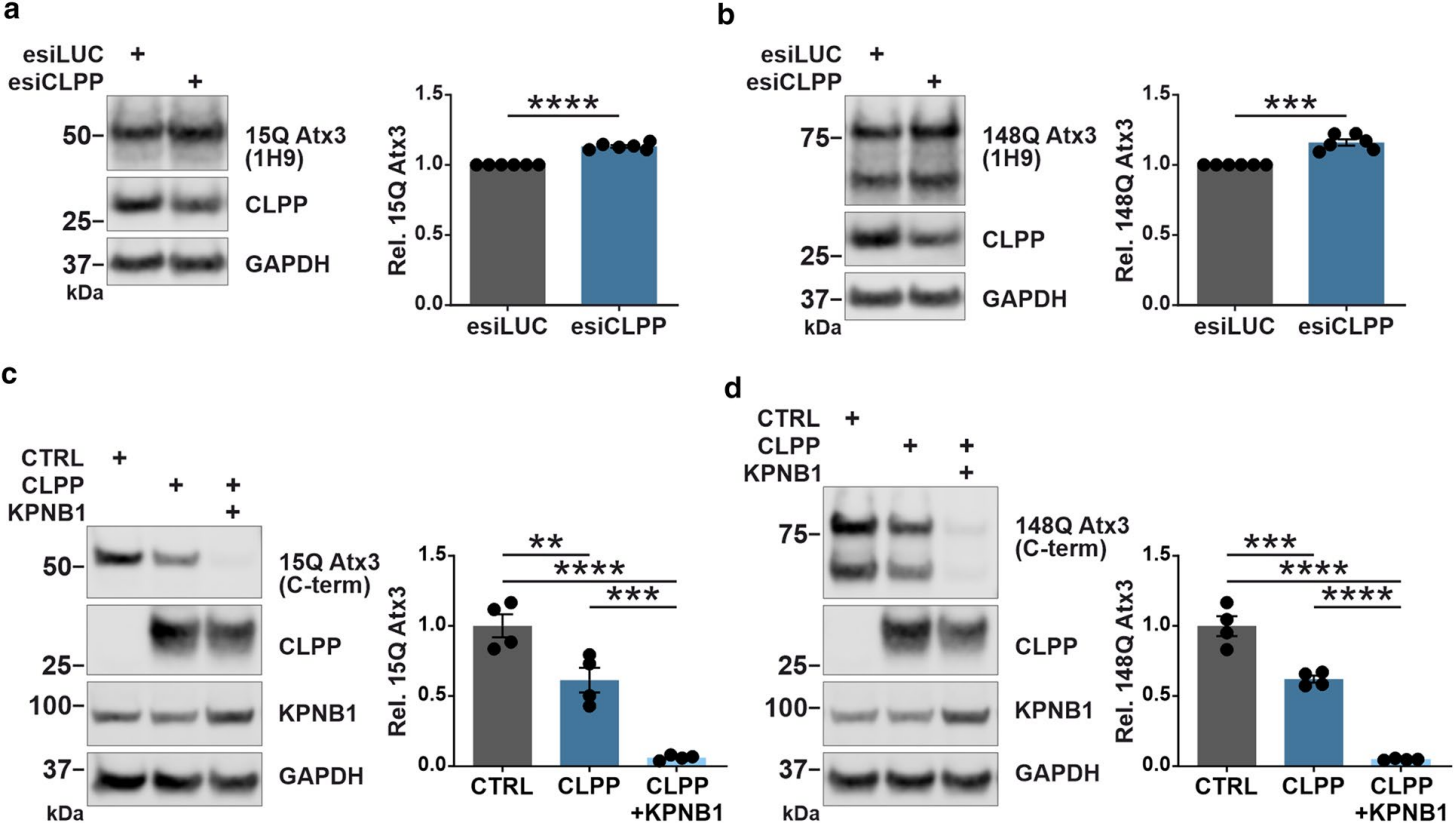
CLPP modulation affects ataxin-3 protein levels. **a**,** b** Western blot analysis reveals that knockdown of CLPP is accompanied by an increase in protein levels of both wild-type (15Q) and polyQ-expanded (148Q) ataxin-3. *ATXN3* KO HEK 293T cells were cotransfected with either 15Q or 148Q ataxin-3 and esiCLPP or esiLUC (control) for 72 h. GAPDH was used as loading control. **a**, *n* = 6, one sample *t*-test, *p* < 0.0001; **b**, *n* = 6, one sample *t*-test, *p* = 0.0009. **c**,** d** CLPP overexpression reduces both 15Q and 148Q ataxin-3 protein levels and its effect is enhanced by KPNB1 overexpression. *ATXN3* KO HEK 293T cells were cotransfected with either 15Q or 148Q ataxin-3 and CLPP or both CLPP and KPNB1 constructs for 72 h. GAPDH was applied as loading control. **c**, *n* = 4, one-way ANOVA with Tukey’s post-test, CTRL *vs* CLPP, *p* = 0.0089; CTRL *vs* CLPP + KPNB1, *p* < 0.0001; CLPP *vs* CLPP + KPNB1, *p* = 0.0009; d, *n* = 4, one-way ANOVA with Tukey’s post-test, CTRL *vs* CLPP, *p* = 0.0004; CTRL *vs* CLPP + KPNB1, *p* < 0.0001; CLPP *vs* CLPP + KPNB1, *p* < 0.0001. *CTRL* empty vector, *C-term* C-terminal, *Rel.* relative. Values are displayed as means ± SEM. **p ≤ 0.01; ***p ≤ 0.001; ****p ≤ 0.0001

As CLPP is a nuclear encoded mitochondrial protease involved in the degradation of unfolded and misfolded proteins [51], we speculated that the reduction of ataxin-3 protein levels in KPNB1 overexpressing cells might be directly associated with the activation of CLPP. Therefore, we validated our hypothesis by knockdown of CLPP using specific esiRNA (esiCLPP) in KPNB1 overexpressing cells. Intriguingly, knockdown of CLPP, as achieved by 47% in esiCLPP-transfected cells, counteracted the reduction of full-length 15Q and 148Q ataxin-3 protein levels (Fig. 6e, f). Filter retardation assay demonstrated an elevated polyQ-expanded ataxin-3 aggregation upon CLPP knockdown in KPNB1 overexpressing cells in comparison to control cells cotransfected with esiLUC (Fig. 6g). To further validate our findings, CLPP was knocked down in 15Q and 148Q ataxin-3 expressing cells, which led to a significant increase in ataxin-3 protein levels (Fig. 7a, b). Conversely, overexpression of CLPP reduced protein levels of both 15Q and 148Q ataxin-3, and this effect was boosted upon additional overexpression of KPNB1 (Fig. 7c, d). These results reinforce the idea that modulation of ataxin-3 by KPNB1 overexpression is likely mediated by the activation of mitochondrial protease CLPP.

### MJD mouse models and iPSCs of MJD patients exhibit reduced KPNB1 protein levels

**Fig. 8:**
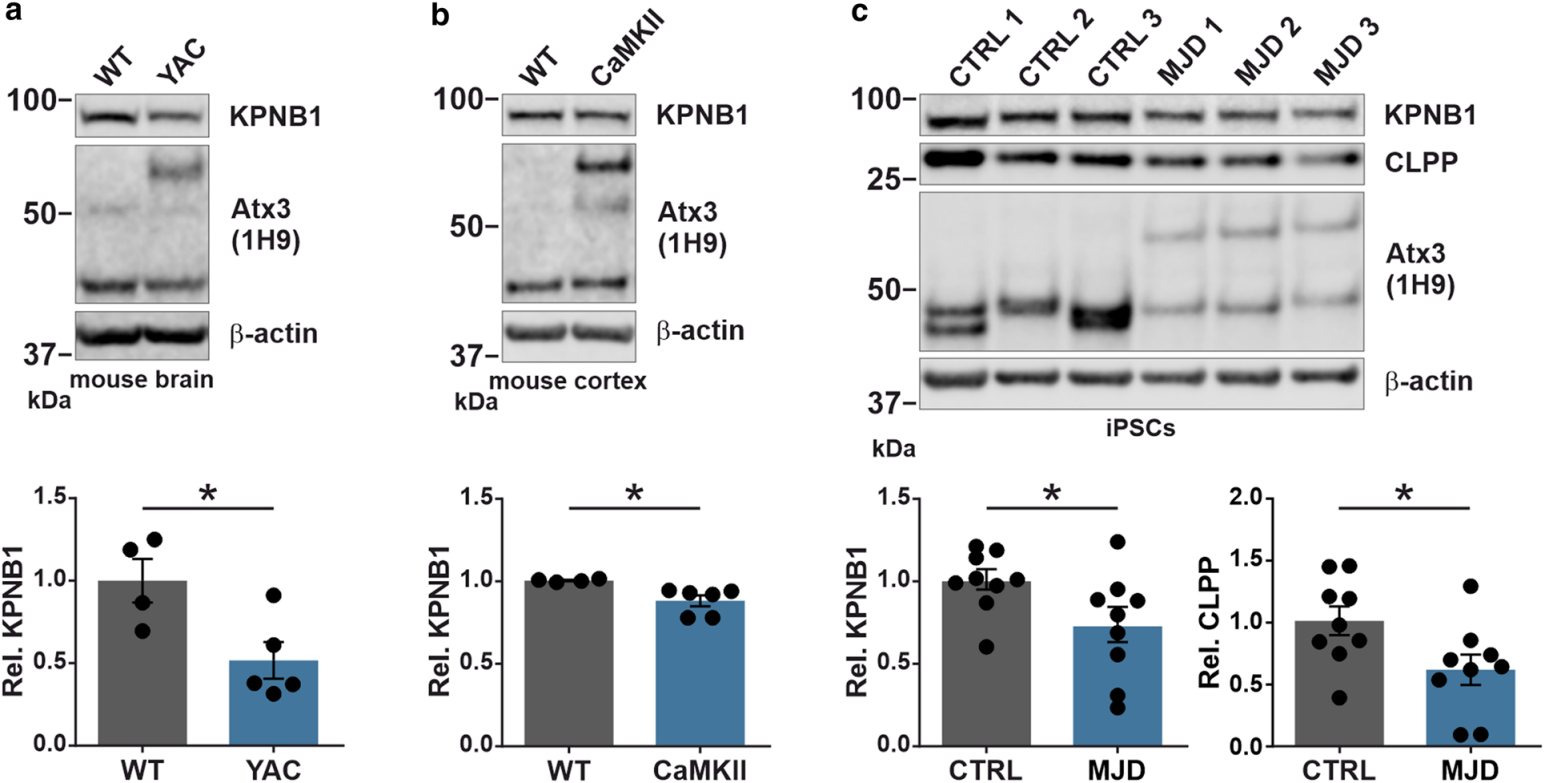
MJD transgenic mouse models and iPSCs of MJD patients exhibit reduced KPNB1 protein levels. **a** Western blot analysis of whole brain protein extracts of 15-month-old YAC transgenic mice indicates a significant reduction of KPNB1 protein levels compared with controls. β-actin was used as loading control. *n* = 4–5, unpaired *t*-test, *p* = 0.0257. **b** Western blot analysis of cortical lysates of 5-month-old CaMKII/MJD77 transgenic mice confirms decreased protein levels of KPNB1 in comparison to controls. β-actin was applied as loading control. *n* = 4–6, unpaired *t*-test, *p* = 0.0175. **c** Western blot analysis of induced pluripotent stem cells (iPSCs) from three MJD patients and three healthy controls demonstrates a reduction of both KPNB1 and CLPP protein levels. β-actin was applied as loading control. *n* = 3 replicates of 3 patients and 3 controls, unpaired *t*-test, KPNB1, *p* = 0.0410; CLPP, *p* = 0.0325. *WT* wild-type, *iPSCs* induced pluripotent stem cells, *CTRL* healthy control, *Rel.* relative. Values are displayed as means ± SEM. *p ≤ 0.05

As we demonstrated KPNB1 as an important modulator of wild-type and polyQ-expanded ataxin-3 protein levels, we sought to investigate its endogenous levels in animal models of MJD. For this purpose, we examined KPNB1 protein levels in the cerebral cortex and whole brain of 5-month-old CaMKII/MJD77 and 15-month-old YAC transgenic mice, respectively. Intriguingly, western blot analysis showed a reduction in KPNB1 protein levels in both MJD mouse models compared with respective controls (Fig. 8a, b). Subsequently, further assessment of KPNB1 and CLPP protein levels was conducted using a patient-derived induced pluripotent stem cell (iPSC) model of MJD. Our findings indicated that both KPNB1 and CLPP protein levels are decreased in MJD patient-derived iPSCs in comparison to healthy controls (Fig. 8c). Investigation of neuronal tissue from two different MJD mouse models and iPSCs of MJD patients laid emphasis on a pathological dysregulation of KPNB1 and CLPP in MJD.

**Fig. 9:**
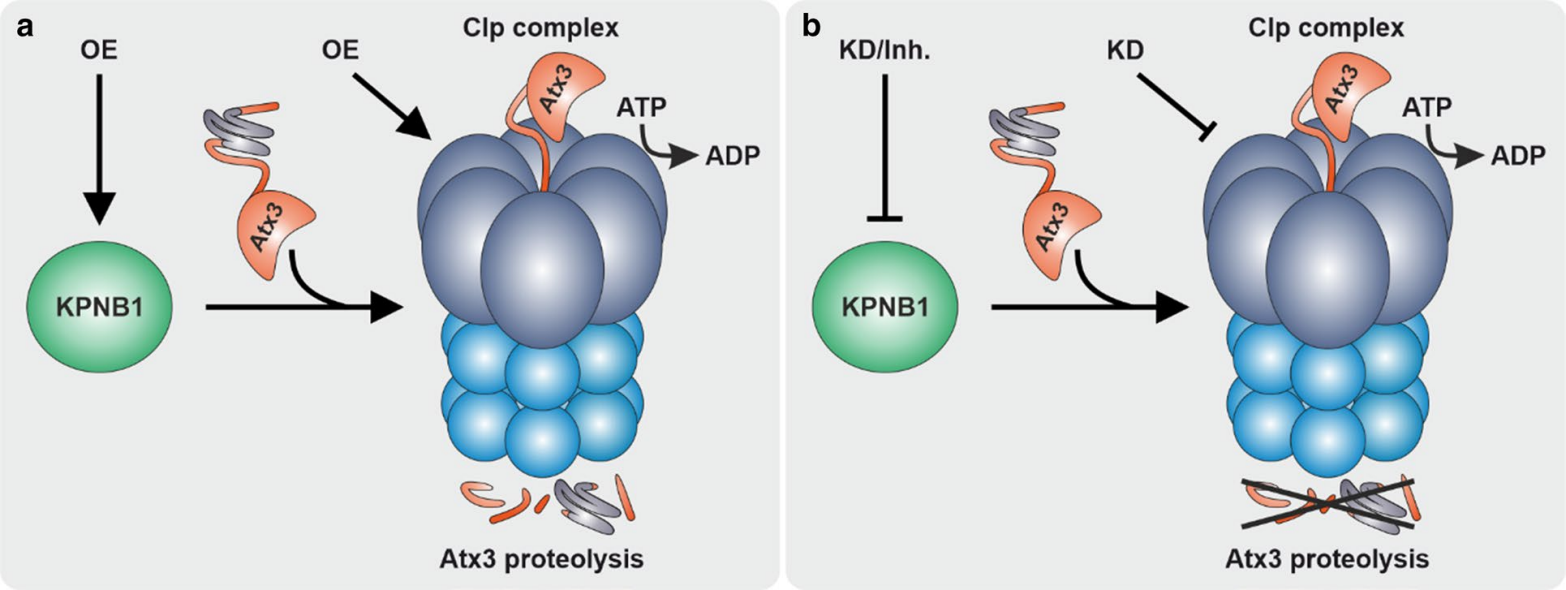
Schematic overview of the proposed ataxin-3 degrading pathway mediated by KPNB1 and CLPP. **a** KPNB1 overexpression activates mitochondrial protease CLPP, leading to the degradation and reduction of ataxin-3 protein levels. Likewise, overexpression of CLPP has a comparable effect on ataxin-3 protein levels. Cleavage of peptides by the protease component of the Clp complex is an ATP-dependent process [76]. **b** KPNB1 knockdown and pharmacological inhibition as well as CLPP knockdown prevent ataxin-3 degradation via the proposed pathway. *OE* overexpression, *KD* knockdown, *Inh.* inhibition

Taken together, we demonstrate that KPNB1 is a modulator of ataxin-3 protein levels, cleavage, and aggregation. Our findings highlight that KPNB1 overexpression lowers ataxin-3 protein levels likely via activation of the mitochondrial protease CLPP (Fig. 9).

## Discussion

The appropriate subcellular localization of proteins is essential for their physiological functions and the maintenance of cellular homeostasis [52]. In recent years, there has been a growing appreciation for the idea that dysregulation of the nucleocytoplasmic transport machinery plays a critical role in the pathogenesis of various neurodegenerative diseases. A close relationship between perturbed nuclear pore complexes (NPCs) and pathology of HD and amyotrophic lateral sclerosis (ALS) has been demonstrated [53, 54]. Apart from this, a defective nucleocytoplasmic transport has been indicated in Alzheimer’s disease (AD) [55], Parkinson’s disease (PD) [56], frontotemporal dementia (FTD) [57], and SCA1 [58].

The preferential accumulation of polyQ-expanded proteins in the nucleus of neurons and the formation of intranuclear inclusions are the pathological hallmarks of MJD and other polyQ diseases, leading to neurotoxicity [59]. It has been suggested that the intrinsically low activity of neuronal nuclear ubiquitin–proteasome system (UPS) may account for the nuclear accumulation of polyQ-expanded proteins [60], even though the precise mechanisms remained enigmatic. In this regard, evaluation of the nucleocytoplasmic transport in MJD and other polyQ diseases might unravel its pathological implication and therapeutic targetability.

Previous investigations by our laboratory revealed KPNA3 as a key player in nucleocytoplasmic transport of ataxin-3 and manifestation of symptoms in MJD animal models [27], encouraging us to study the relevance of other karyopherins on ataxin-3 subcellular localization as well. Here, we demonstrated a physical interaction of KPNA3 and KPNB1 with ataxin-3. However, our further analysis of KPNB1 showed no effect of its modulation on the subcellular distribution of either wild-type or polyQ-expanded ataxin-3. Instead, we observed a marked reduction of wild-type and polyQ-expanded ataxin-3 soluble protein levels and enhancement of ataxin-3 cleavage upon KPNB1 overexpression, which is more prominent in wild-type ataxin-3. This effect was not compromised by mutating the FxFG binding site of KPNB1, indicating a nuclear transport function-independent mechanism behind ataxin-3 reduction. Conversely, KPNB1 knockdown and its pharmacological inhibition using importazole resulted in an increase of ataxin-3 protein levels. Given that KPNB1 modulates both wild-type and polyQ-expanded ataxin-3, it seems to be a polyQ-independent and therefore physiological mechanism.

Furthermore, we analyzed the nature of the observed KPNB1-induced ataxin-3 fragments. Ataxin-3 cleavage products have been detected both in the brains of MJD patients and MJD mouse models [18, 61]. Both caspases and calpains have been associated with proteolysis of ataxin-3 in the MJD context [62]. Also, ataxin-3 has been described as a substrate for autophagic and proteasomal degradation [46, 47]. However, we could not substantiate an involvement of either class of proteases in ataxin-3 fragmentation upon KPNB1 overexpression, or the autophagic or proteasomal machineries, indicating the contribution of a yet unknown proteolytic enzyme.

We postulated that modulating KPNB1 might influence the aggregation of polyQ-expanded ataxin-3 as well as its soluble levels. Accordingly, KPNB1 overexpression diminished aggregation and improved the cell viability, whereas KPNB1 knockdown and pharmacological inhibition had the opposite effect on ataxin-3 aggregates. As we observed a modulation of full-length ataxin-3 upon KPNB1 overexpression or knockdown, we concluded that the changes in polyQ-expanded ataxin-3 aggregation were due to its altered soluble levels, as the concentration of polyQ-expanded ataxin-3 has direct consequences for aggregate formation [63].

Interestingly, we observed reduced KPNB1 protein levels in neuronal tissue of two different MJD transgenic mouse models and reduction of both KPNB1 and CLPP protein levels in iPSCs derived from MJD patients, which provides evidence for a pathological dysregulation of KPNB1 and CLPP in MJD.

To further dissect the KPNB1-mediated effects on ataxin-3 levels, we performed label-free quantitative proteomics followed by pathway analysis and discovered a potential activation of the mitochondrial protease CLPP in KPNB1 overexpressing cells. CLPP is a serine protease which plays a pivotal role in the degradation and regulation of synthesis of proteins that are related to cellular metabolic pathways, such as electron transport chain. Nonetheless, little is known about the implication of this mitochondrial protease in cellular pathways and mechanisms [64, 65]. We could corroborate our findings by esiRNA-mediated knockdown of CLPP in KPNB1 overexpressing cells, which led to the rescue of soluble and aggregated forms of ataxin-3. In addition, CLPP overexpression reduced ataxin-3 protein levels, which was further enhanced by KPNB1 cotransfection in these cells. Previous investigations have shown that loss of CLPP is implicated in α-synuclein-associated pathology in induced neurons derived from PD patients. Conversely, overexpression of CLPP inhibits the accumulation of S129-phosphorylated α-synuclein, leading to the promotion of neuronal morphology in these cells [66]. The potential site of the interaction between ataxin-3 and the mitochondrially localized CLPP remains obscure. However, both wild-type and polyQ-expanded ataxin-3 have been shown to be associated with or localized in mitochondria [63, 67]. Some mitochondrial proteins have been identified as interaction partners of wild-type and/or polyQ-expanded ataxin-3, such as cytochrome C oxidase subunit NDUFA4 (NDUFA4), succinate dehydrogenase complex subunits A and B (SDHA and SDHB), and cytochrome C oxidase assembly factor 7 (COA7) [67, 68].

Aside from its role as a nuclear transport protein, KPNB1 has been described as a multifunctional protein involved in cell cycle control, mitosis and replication, promoting endoplasmic-reticulum-associated degradation (ERAD), and also functioning as a chaperone which prevents aggregation of some proteins [69–71]. Moreover, dysregulation of KPNB1 has been linked to several types of cancer [72–75]. The herein described activation of a mitochondrial protease such as CLPP, and thereby the induction of proteolytic turnover would represent a new addition to the functional repertoire of KPNB1.

In sum, our study reveals KPNB1 as a new putative modulator of ataxin-3, which triggers proteolytic degradation via mitochondrial protease CLPP, and consequently, lowers aggregate levels of the polyQ-expanded protein. Further analysis will better assess the therapeutic value of targeting KPNB1 or CLPP not only in MJD but also in other neurodegenerative proteopathies.

### Supplementary Information

Below is the link to the electronic supplementary material.

Supplementary file1 (DOCX 807 KB)

## Data Availability

Not applicable.
