# Supplementary material for: KPNB1 modulates the Machado–Joseph disease protein ataxin-3 through activation of the mitochondrial protease CLPP
Source: Cell Mol Life Sci. 2022 Jul 6;79(8):401. doi: 10.1007/s00018-022-04372-5 (PMC9259533; doi:10.1007/s00018-022-04372-5)
Supplement: Supplementary file 1 — Supplementary file1 (DOCX 807 KB) [file 18_2022_4372_MOESM1_ESM.docx]

**Supplementary Information**

**KPNB1 modulates the Machado-Joseph disease protein ataxin-3 through activation of the mitochondrial protease CLPP**

Mahkameh Abeditashi^1,2,3^, Jonasz Jeremiasz Weber^1,2,4^, Priscila Pereira Sena^1,2,3^, Ana Velic^5^, Maria Kalimeri^1,2^, Rana Dilara Incebacak Eltemur^1,2,4^, Jana Schmidt^1,2^, Jeannette Hübener-Schmid^1,2^, Stefan Hauser^6,7^, Boris Macek^5^, Olaf Riess^1,2^, and Thorsten Schmidt^1,2*^

^1^ Institute of Medical Genetics and Applied Genomics, University of Tübingen, Tübingen 72076, Germany

^2^ Centre for Rare Diseases, University of Tübingen, Tübingen 72076, Germany

^3^ Graduate Training Centre of Neuroscience, University of Tübingen, Tübingen 72076, Germany

^4^ Department of Human Genetics, Ruhr University Bochum, Bochum 44801, Germany

^5^ Proteome Center Tübingen, University of Tübingen, Tübingen 72076, Germany

^6^ German Center for Neurodegenerative Diseases (DZNE), Tübingen 72076, Germany

^7^ Department of Neurology and Hertie Institute for Clinical Brain Research, University of Tübingen, Tübingen 72076, Germany

* **Corresponding author.** Phone: +49 7071 29 72277

Email: Thorsten.Schmidt@med.uni-tuebingen.de

**Supplementary Information**


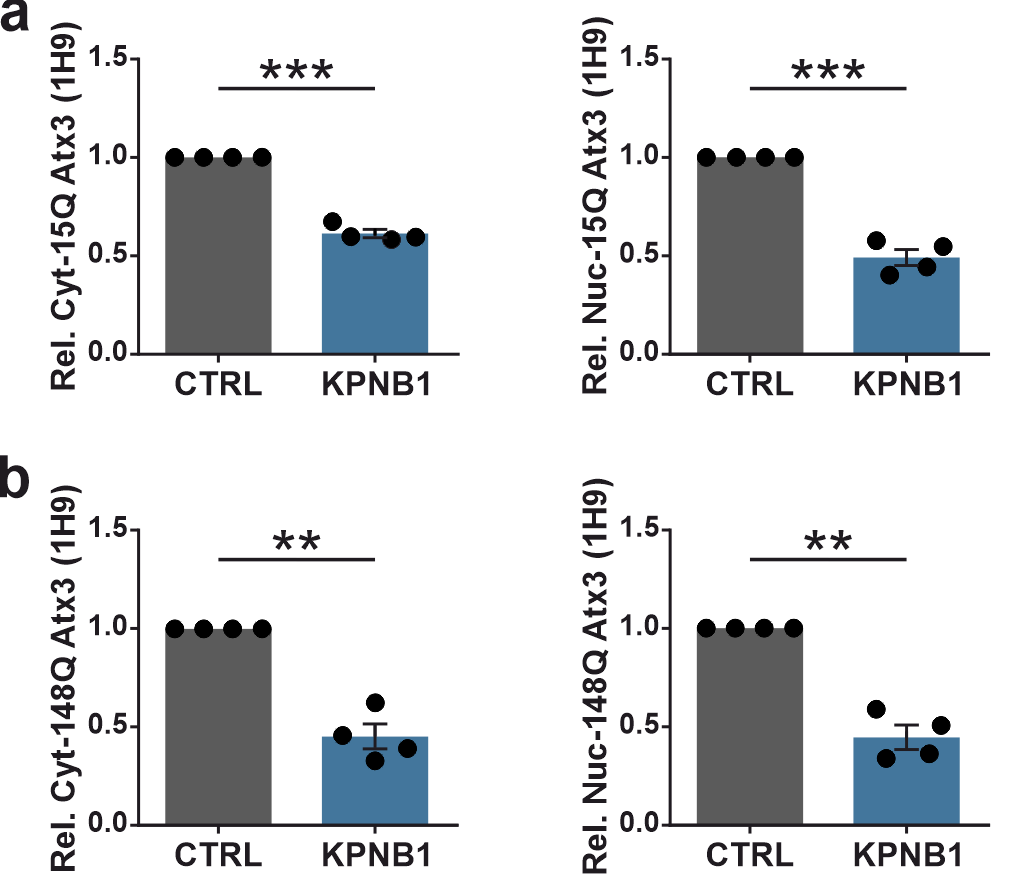


**Fig. S1 Reduction of both cytoplasmic and nuclear ataxin-3 in KPNB1 overexpressing cells.** **a**, **b** Diagrams illustrate the quantifications of nucleocytoplasmic fractionation assay. Both cytoplasmic and nuclear levels of either wild-type (15Q) or polyQ-expanded (148Q) ataxin-3 decrease upon KPNB1 overexpression. a, *n* = 4, Cyt-15Q, one sample *t*-test, *p*= 0.0003; Nuc-15Q, one sample *t*-test, *p*= 0.0012; b, *n* = 4, Cyt-148Q, one sample *t*-test, *p*= 0.0033; Nuc-148Q, one sample *t*-test, *p*= 0.0027. CTRL = empty vector; Cyt = cytoplasmic fraction; Nuc = nuclear fraction; Rel. = relative. Values are displayed as means ± SEM. ***p*≤ 0.01; ****p*≤ 0.001.


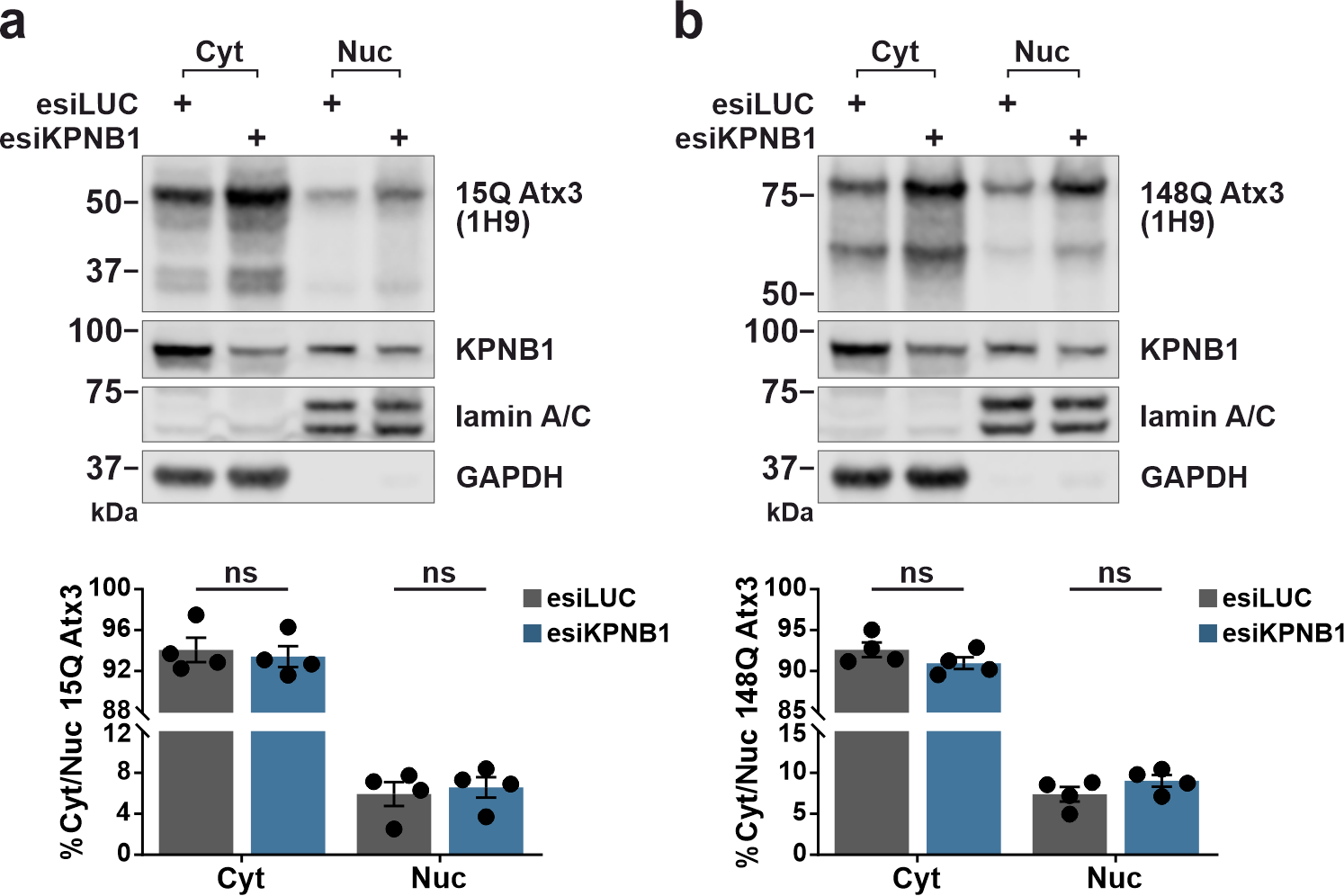


**Fig. S2 KPNB1 knockdown does not modulate the intracellular localization of ataxin-3.** **a**, **b** Nucleocytoplasmic fractionation assay indicates an increase of both cytoplasmic and nuclear wild-type (15Q) and polyQ-expanded (148Q) ataxin-3 upon KPNB1 knockdown. However, no alteration was observed in the subcellular localization of ataxin-3. *ATXN3* KO HEK 293T cells were cotransfected with either 15Q or 148Q ataxin-3, and esiKPNB1 or esiLUC as control. 72 h post-transfection, nucleocytoplasmic fractionation assay was performed and followed by western blot analysis. The diagrams illustrate the ratio of nuclear and cytoplasmic ataxin-3 as percentage. GAPDH and lamin A/C were applied as cytoplasmic and nuclear loading controls, respectively. a, *n* = 4, Cyt/Nuc, unpaired *t*-test, *p*= 0.6901; b, *n* = 4, Cyt/Nuc, unpaired *t*-test, *p*= 0.2029. Cyt = cytoplasmic fraction; Nuc = nuclear fraction. Values are displayed as means ± SEM. ns = not significant.


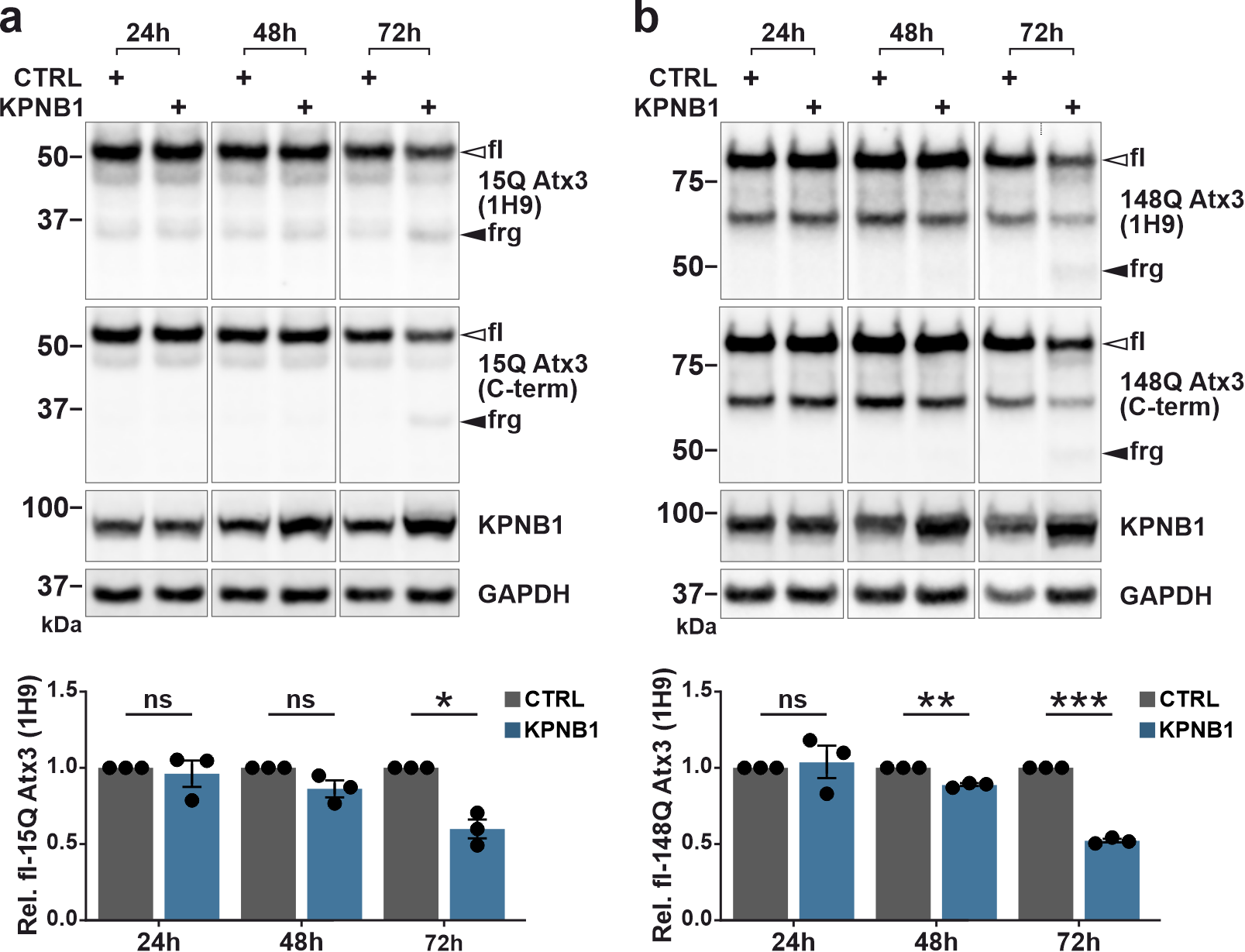


**Fig. S3 Decrease of ataxin-3 protein levels upon KPNB1 overexpression is time-dependent. a**, **b** 15Q or 148Q ataxin-3 overexpressing HEK 293T cells were cotransfected with either KPNB1 or empty vectors for different durations (24 h, 48 h, and 72 h). Western blot analysis shows that the reduction of both 15Q and 148Q ataxin-3 protein levels is promoted by increasing the time of KPNB1 overexpression and it becomes more obvious 72 h post-transfection. Furthermore, accumulation of ataxin-3 fragments is apparent after 72 h KPNB1 overexpression compared with 24 h and 48 h. Black arrowheads indicate ataxin-3 fragments. Ataxin-3 was detected by 1H9 and C-terminal antibodies. GAPDH was used as loading control. a, *n* = 3, one sample *t*-test, CTRL *vs* KPNB1 (24 h), *p*= 0.7083; CTRL *vs* KPNB1 (48 h), *p*= 0.1225; CTRL *vs* KPNB1 (72 h), *p*= 0.0229; b, *n* = 3, one sample *t*-test, CTRL *vs* KPNB1 (24 h), *p*= 0.7608; CTRL *vs* KPNB1 (48 h), *p*= 0.0065; CTRL *vs* KPNB1 (72 h), *p*= 0.0005. CTRL = empty vector; fl = full-length; frg = fragment; C-term = C-terminal; Rel. = relative. Values are displayed as means ± SEM. ns = not significant; **p*≤ 0.05; ***p*≤ 0.01; ****p*≤ 0.001.


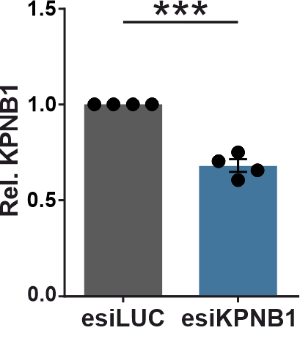


**Fig. S4 Reduction of KPNB1 protein levels using esiKPNB1.** KPNB1 knockdown using esiKPNB1 indicates a 32% effectiveness in lowering protein levels compared with control (esiLUC). *n* = 4, one sample *t*-test, *p*= 0.0023. Rel. = relative. Values are displayed as means ± SEM. ****p*≤ 0.001.


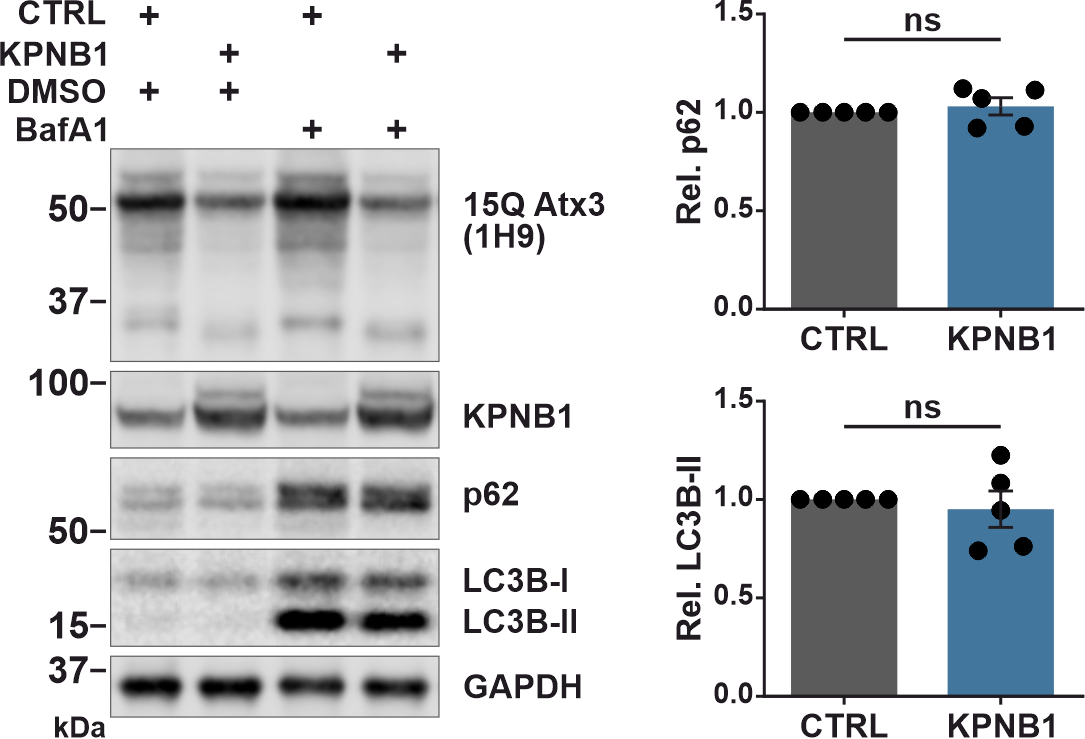


**Fig. S5 KPNB1 overexpression does not affect autophagy.** Western blot analysis demonstrates no alteration in the levels of autophagic markers, p62 and LC3B-II, upon KPNB1 overexpression. *ATXN3* KO HEK 293T cells cotransfected with 15Q ataxin-3 and KPNB1 or empty vectors were incubated with 50 nM autophagy inhibitor bafilomycin A1 or DMSO for 16 h prior to harvesting. The diagrams illustrate p62 and LC3B-II protein levels in bafilomycin A1 treated cells. GAPDH was applied as loading control. *n* = 5, p62, one sample *t*-test, *p*= 0.5239; LC3B-II, one sample *t*-test, *p*= 0.6233. CTRL = empty vector; BafA1 = bafilomycin A1; Rel. = relative. Values are displayed as means ± SEM. ns = not significant.

**Table S1** Label-free quantitative proteomics indicated 15 common proteins between wild-type (15Q) and polyQ-expanded (148Q) ataxin-3 expressing cells which were significantly upregulated or downregulated upon KPNB1 overexpression. FC = fold-change.

| **Protein name** | **Gene name** | **-Log_10_ *p*-value** | **Log_2_ FC** | **-Log_10_ *p*-value** | **Log_2_ FC** |
| --- | --- | --- | --- | --- | --- |
|  |  | **15Q ataxin-3** | | **148Q ataxin-3** | |
| Importin subunit alpha-1 | *KPNA2* | 1.8884 | 0.2815 | 1.9011 | 0.3919 |
| Replication protein A 14 kDa subunit | *RPA3* | 1.6005 | 0.2699 | 2.3108 | 0.1511 |
| Peptidyl-prolyl cis-trans isomerase A;Peptidyl-prolyl cis-trans isomerase A, N-terminally processed;Peptidyl-prolyl cis-trans isomerase | *PPIA* | 2.1972 | 0.1424 | 1.6160 | 0.1933 |
| Filamin-B | *FLNB* | 1.5187 | -0.0694 | 2.3212 | -0.1163 |
| Succinyl-CoA ligase [GDP-forming] subunit beta, mitochondrial | *SUCLG2* | 2.0348 | -0.0959 | 1.7882 | -0.1858 |
| Aspartate--tRNA ligase, mitochondrial | *DARS2* | 1.8837 | -0.1395 | 1.4139 | -0.2035 |
| Nuclear pore complex protein Nup155 | *NUP155* | 1.4146 | -0.1514 | 1.6854 | -0.1444 |
| 3-hydroxyisobutyryl-CoA hydrolase, mitochondrial | *HIBCH* | 1.3204 | -0.1582 | 1.8878 | -0.1885 |
| Dual specificity mitogen-activated protein kinase kinase 2 | *MAP2K2* | 1.3529 | -0.1644 | 1.3333 | -0.1988 |
| Isocitrate dehydrogenase [NADP], mitochondrial | *IDH2* | 2.3184 | -0.2038 | 2.0786 | -0.2143 |
| Farnesyl pyrophosphate synthase | *FDPS* | 1.7910 | -0.2457 | 1.7474 | -0.1717 |
| Angiomotin | *AMOT* | 1.4788 | -0.2578 | 2.1617 | -0.2684 |
| Erlin-2 | *ERLIN2* | 1.3274 | -0.2602 | 1.7787 | -0.2581 |
| Lanosterol 14-alpha demethylase | *CYP51A1* | 1.3446 | -0.2841 | 2.9263 | -0.1569 |
| Protein FAM98B | *FAM98B* | 1.7179 | -0.3313 | 2.5785 | -0.4639 |

**Table S2** Ingenuity Pathway Analysis (IPA) predicted activation of the mitochondrial protease CLPP upon KPNB1 overexpression, as proteins which are associated with the activation of CLPP were downregulated significantly. FC = fold-change.

| **Protein name** | **Gene name** | **-Log_10_ *p*-value** | **Log_2_ FC** |
| --- | --- | --- | --- |
| Pyruvate dehydrogenase E1 component subunit alpha, mitochondrial | *PDHA1* | 1.8413 | -0.2333 |
| Isocitrate dehydrogenase [NADP], mitochondrial | *IDH2* | 2.8281 | -0.2090 |
| Medium-chain specific acyl-CoA dehydrogenase, mitochondrial | *ACADM* | 2.5170 | -0.2022 |
| Aldehyde dehydrogenase, mitochondrial | *ALDH2* | 2.1147 | -0.2008 |
| Dihydrolipoyllysine-residue succinyltransferase component of 2-oxoglutarate dehydrogenase complex, mitochondrial | *DLST* | 1.3514 | -0.1863 |
| Succinate dehydrogenase [ubiquinone] flavoprotein subunit, mitochondrial | *SDHA* | 1.6132 | -0.1838 |
| 2,4-dienoyl-CoA reductase, mitochondrial | *DECR1* | 1.5326 | -0.1742 |
| Aconitate hydratase, mitochondrial | *ACO2* | 1.4078 | -0.1522 |
| Mitochondrial 2-oxoglutarate/malate carrier protein | *SLC25A11* | 1.8609 | -0.1324 |
| Pyruvate dehydrogenase E1 component subunit beta, mitochondrial | *PDHB* | 1.8095 | -0.1257 |
